# School-based behavioral intervention to reduce the habit of smokeless tobacco and betel quid use in high-risk youth in Karachi: A randomized controlled trial

**DOI:** 10.1371/journal.pone.0206919

**Published:** 2018-11-02

**Authors:** Azmina Hussain, Sidra Zaheer, Kashif Shafique

**Affiliations:** 1 Dr. Ishrat-ul-Ibad Khan Institute of Oral Health Sciences, Dow University of Health Sciences, Gulzar e Hijri, Karachi, Pakistan; 2 School of Public Health, Dow University of Health Sciences, Gulzar e Hijri, Karachi, Pakistan; 3 Institute of Health and Wellbeing, Public Health, University of Glasgow, Glasgow, United Kingdom; TNO, NETHERLANDS

## Abstract

There have been recent surges in the use of smokeless tobacco (SLT) and betel quid (BQ) chew among adolescents in South East Asian countries, with an increase, on average, of 7% to 15% between 2004 and 2013, necessitating interventional investigations to modify this behavior. The current intervention was aimed towards changing adolescents’ perceptions regarding the harmful effects of SLT and BQ use and encouraging them to quit. This randomized control trial involved 2140 adolescents from 26 private and public-sector schools in Karachi, Pakistan. After randomization, 1185 individuals were placed in the intervention group and administered a behavior changing intervention (BCI), while 955 individuals constituted the control group. A generalized estimating equation was employed to measure differences in repeated measures for both groups. The beta coefficients were reported after adjusting the covariates with the 95% confidence interval, and the p-value was considered significant at <0.050. Cohen’s d was employed to report the effect size of the intervention. The BCI resulted in a 0.176-unit (95% CI 0.078–0.274, p-value <0.001) increase in knowledge scores regarding the health hazards of SLT and BQ, a 0.141-unit (95% CI 0.090–0.192, p-value <0.001) increase in use perception scores, and a 0.067-unit (95% CI 0.006–0.129, p-value 0.031) increase in quit perception scores in the intervention group compared with those in the control group. A knowledge related module (p-value 0.024) and quit preparation module (p-value 0.005) were found to be helpful by adolescents in either changing their perceptions regarding SLT and/or BQ chew use or in quitting. The role of BCI is promising in improving adolescents’ knowledge and changing their perceptions in a positive manner regarding their harmful SLT and BQ use. Convincing results may be achieved if interventions are tailored, with an emphasis on the identification of the products that are used by adolescents in addition to highlighting their ill effects and how students may manage to quit them. If included in the schools’ curricula, this BCI method may help in developing schools that are free of SLT and BQ use.

**Trial registration:** ClinicalTrials.gov NCT03488095.

## Introduction

Smokeless tobacco (SLT) is an extensively used term that includes various types of tobacco products that are either consumed orally or are taken via the nose [1]. In approximately 115 countries, more than 300 million adults consume SLT in various forms [2]; among these, the majority of the consumers (89%) are concentrated in South Asian countries [3, 4]. During the years 2004 to 2013, the chewing of SLT among adolescents substantially increased from approximately 7% to 15% in the South East Asian Region (in Bhutan from 7.4% in 2004 to 21.6% in 2013; in Nepal from 6.1% to 16.2% between 2007 and 2011; in Myanmar, the increase in SLT chews ranged from 6.5% to 9.8% between the years 2004 and 2011) [4]. SLT use is accepted in South Asian countries as an appropriate component of the cultural and social norms of society[5]. When young individuals start using SLT (this habit of chewing SLT and BQ commences at a very young age of nearly 15 years[6]), they become dependent on it (as the former is the fourth most commonly abused substance after caffeine, alcohol and nicotine) because of their simultaneous stimulant and relaxant effects [4, 7–9].

Nicotine and nitrosamines that are present in SLT cause oral and oropharyngeal cancers and are the cause of more than 0.25 million deaths globally [2]. Betel quid (BQ) is also a type of SLT that contains areca nut, which may or may not have tobacco[4, 10]. SLT, betel quid and areca nut alone or in combination are known to increase the incidence of oral squamous cell carcinoma irrespective of their tobacco content [4, 10–12], and they can cause oral potentially malignant lesions [4, 13] and coronary heart diseases [4, 14].

A Cochrane review greatly supported the effectiveness of behavioral interventions in helping SLT and BQ chewers quit this fatal habit [15, 16]. Nonetheless, a Cochrane review that was updated in 2015 on the interventions to promote cessation of SLT chewing reported (from 17 behavioral interventions)[15] that the confidence in the effect sizes of interventions was limited since it was not clear which constituents of the interventions influenced their impact. Gansky reported dubious effects of BCI since the SLT cessation effects were more pronounced in the control group due to strong legislation against SLT use in the western world [17]. Walsh *et al*. [18] randomized 44 schools either to an intervention or control group and suggested that BCI was effective in both users and nonusers since SLT users were at an early stage of habit-building and that they used less addictive products than South Asian individuals.

A systematic review and meta-analysis strongly suggested that since SLT use has a very strong cultural backdrop attached to South East Asian countries with scant awareness available regarding its deleterious effects on oral health along with low literacy rates, culturally designed BCI will likely play a positive role in controlling SLT and BQ chewing among a high-risk group of 13–15 year-olds [19]. Since the increasing oral cancer trend in South Asian countries is due to the use of SLT [4], which is completely avoidable, influencing the use of SLT chew can serve as a promising initiation point to control the incidence of oral cancer and plan primary preventive interventions[19]. A BCI rooted in Asian culture was also developed and tested in Pakistan and the UK in a very small group of 32 adults, but this method determined the efficacy of BCI in curbing the habit of SLT use [20].

Since most of the interventions (inclusive of a Cochrane review on interventions for the cessation of SLT use updated in 2015) were designed and performed in adults living in the western world, it was unrealistic to assume an extrapolation of their effectiveness on adolescents in South Asian countries[21].

Given the increasing disease burden of oral cancer in South Asian countries due to the increasing popularity of SLT consumption, our objective was to develop (a) a BCI that was pertinent to the sociocultural aspects of South Asian SLT users, followed by (b) assessing its efficacy in changing knowledge and perceptions regarding SLT use and cessation in a high risk group of 11–16 year old school-going children in Karachi who attended both private and public-sector schools.

We also aimed to deconstruct our BCI and establish which components of our BCI were most useful and efficient in making an intervention by using self-perceived efficacy reporting from each end-user of the BCI.

## Methodology

### Data source

Karachi is the largest city of Pakistan, with a population of approximately 16 million [22]. The city has been divided into six (6) districts and 18 administrative towns by the City Government of Karachi [23].

### Sampling and study participants

**Fig 1:**
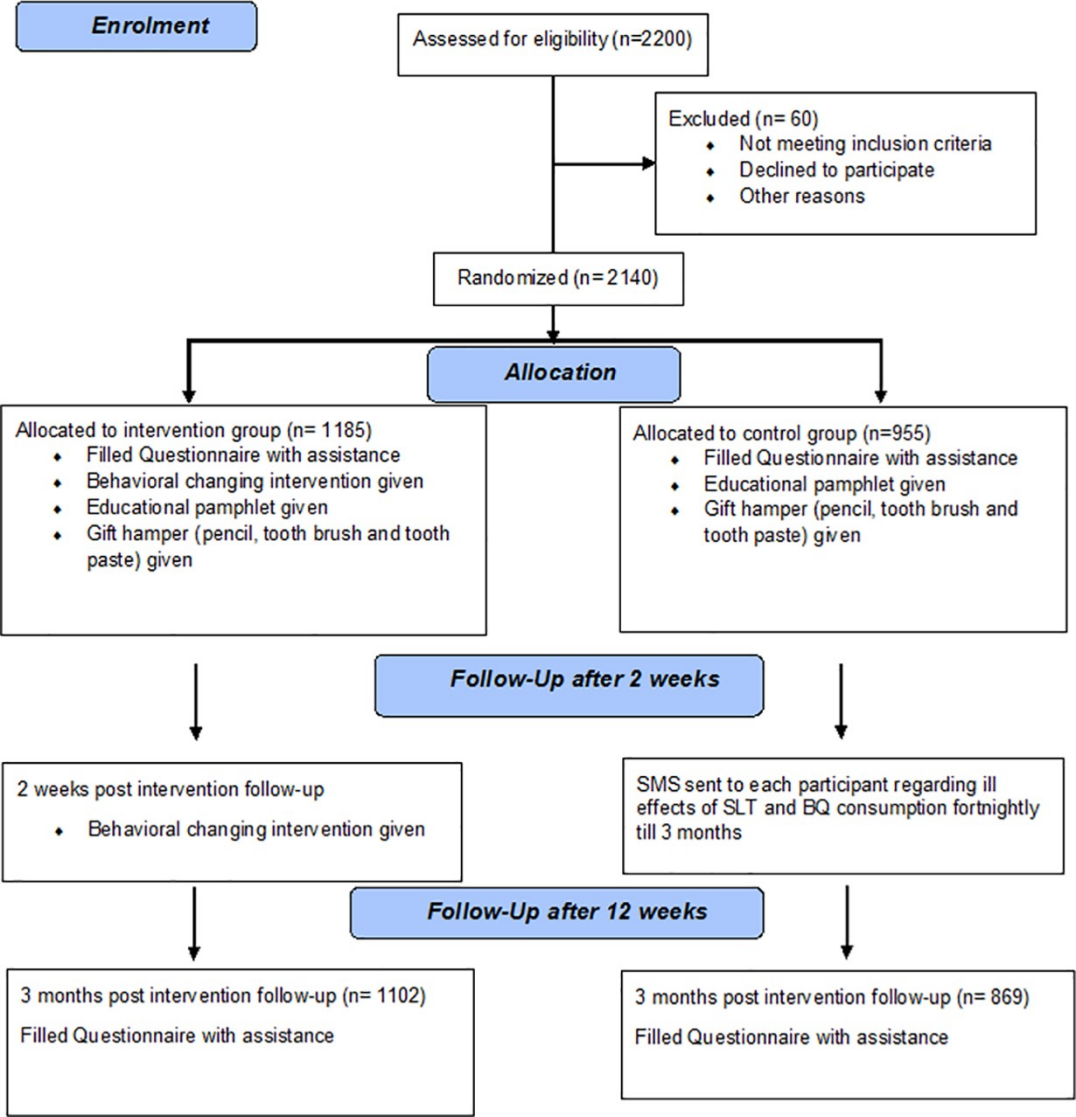
CONSORT flow diagram: Enrolment, allocation, and follow-up[24].

The sampling frame of the current study was the governmental and private schools of Karachi. Cluster sampling was done. From within six districts, 26 clusters (secondary schools) were randomly invited (by using computer-generated random numbers) to participate in the study, ensuring equitable inclusion of each school type (both public and private schools). A decline from an invited school was followed by an invitation to another school from the same district; in addition, a governmental school was selected if another governmental school declined and vice versa. Grade VI-X students (11–16 years old) were randomly recruited (by systematic sampling using class registers) after sorting the consents from the parents and school. Depending upon the size of each class, students who were present in the school on the day of the visit were randomly selected from their classes until a total number of 50–100 students was achieved (Fig 1).

### Sample size calculation

The efficacy of the web-based intervention for all tobacco use cessation was the basis of the sample size calculation for this study. A sample size of 1264 students was calculated in openEpi, using a cohort/RCT formula and a confidence interval (1-alpha) of 95% with 90% power to detect a minimum change of 9% over a control cluster for BCI efficacy in the intervention arm (exposed group vs. unexposed group) [25]. We increased the sample size to 1600, and after factoring in an attrition rate of approximately 35% and rounding it off to the whole number, it was increased to 2200 participants. The cluster size and the number of clusters were calculated manually using equations [26] which resulted in 13 clusters in each arm with 50–100 participants in each cluster, depending on the population size of the school.

### Informed consent and recruitment

The principals of all invited schools were sent the details of the intervention and were requested to consent to their school’s participation in the trial. The principals were also asked to seek permission from parents, and, in the case of any query, the parents were advised to contact the principal investigator via a text message; in return, the principal investigator would call the parents to clarify their issues (since we did not have a toll free number).

It was requested that parents accepted or refused the form and returned it to the school management within a specified timeline.

### Behavior changing intervention (BCI)

The behavior changing intervention (BCI) for the cessation of SLT and betel quid (with or without tobacco) was based on changing the perceptions of the 11–16 year-old school-going children regarding effects of SLT & BQ and assisting them in quitting this life-threating habit. The methodology acquired in designing, developing and assessing the accuracy of this BCI was based on the Medical Research Council framework for complex interventions[27].

The BCI was formulated by using the theoretical behavior changing techniques (BCTs) underpinning the sociocultural norms of South Asian populations that were likely to motivate young users to quit SLT and BQ. The following steps were followed in its development:

1. Recognition and characterization of all key contributing factors that are associated with SLT & BQ use in South Asian Youths and their associated BCTs,
2. Transforming all BCTs into culturally tailored and appropriate behavior changing activities that were not limited to highlighting the adverse and life-threatening outcomes of SLT & BQ use,
3. And assessing the feasibility of the delivery of this approach in young children by a panel of experts who assessed each component of the BCI intervention based on their expertise and termed it as appropriate or inappropriate; only the appropriate components were part of the intervention.

With an aim to influence the youth’s perception regarding discontinuing the use of these products, the most recent taxonomy of BCTs was adapted to translate these key contributing factors that are associated with SLT & BQ use into the BCI by focusing more on oral cancer prevention and/or early detection[28].

This BCI was then designed into an intervention which was culturally sound in South Asian population and kept all cultural sensitivities as a backdrop by a panel of experts who were experienced in designing such activities in South Asian communities. Thus, the harmful effects of the BCI were highlighted without stigmatizing its sociocultural use.

A guide for behavioral support for smoking cessation [29] was kept as a guideline for designing this intervention into pre-quit, quit and post-quit management of withdrawal symptoms.

### The components of BCI included

- Identifying the SLT and BQ products, their harmful effects, and why is it important to quit along with setting a quit date
- Preparing to quit along with managing urges for relapse, and
- Recognizing and managing withdrawal symptoms

All the questions that were asked in the BCI were initially ascertained by the group of experts on the subject and were also tested for their validity in the field by both the enumerator and supervisor. The questions were then communicated to the researcher, after which a final version of the questionnaire was completed. Cronbach's alpha for each component were 0.692 (knowledge), 0.085 (Perception regarding use) and 0.614 (perception regarding quit).

### Intervention and control groups (randomization and concealment)

Schools (clusters) that finally agreed to participate in the intervention were randomly recruited in the intervention and control groups[17] to avoid any selection bias. However, all intervention material was delivered to control clusters at the end of the study to cater to the ethical parameters.

We recruited clusters (schools) in each intervention and control group to avoid any diminution of the intervention outcome to participate in a discussion regarding the intervention among the participants of the same school. Previously, Gansky and colleagues[17] attributed the failure of their intervention to the “spill over” effect due to the contact between athletes in the intervention and control groups.

Both groups’ participants completed pre- and post-intervention questionnaires as described elsewhere. Of 2200 participants, 60 did not provide us with complete information; thus, the final analyses were performed on 2140 participants.

### Duration of the trial

The enrollment of the participants in the study from the school was conducted from April 17, 2016 to April 23, 2016.

The first intervention was conducted during the period of April 25 –May 07, 2016, with simultaneous visits to the control school clusters.

For the intervention clusters, follow-up visits were conducted after 2 weeks (for the second intervention) during the period of May 09–18, 2016

Second follow-up visits for both the intervention and control schools (12 weeks after the first intervention) were done during the period of July 30, 2016 –Aug 12, 2016 with simultaneous visits to the control school clusters.

The trial was registered on clinicaltrial.gov on April 16, 2016 and protocol ID- DUHS/DR-O/2016/116 was generated. *The authors confirm that all on-going and related trials for this intervention are registered*.

### Ethical considerations

This study was critically reviewed by the scientific committee of Dow University of Health Sciences (DUHS), and the ethical parameters were scrutinized by team members of the Institutional Review Board (IRB). After thorough review, ethical permission was granted for this study by IRB-DUHS (Reference Number: IRB-725/DUHS/Approval/2016/219) on April 16, 2016.

### Implementation of BCI

Students in the intervention group were shown a well-structured visual presentation that assessed their perceptions regarding SLT and BQ use, while the controls were not exposed to BCI. The intervention was delivered by trained personnel to avoid any researcher bias (Fig 1)

**Fig 2:**
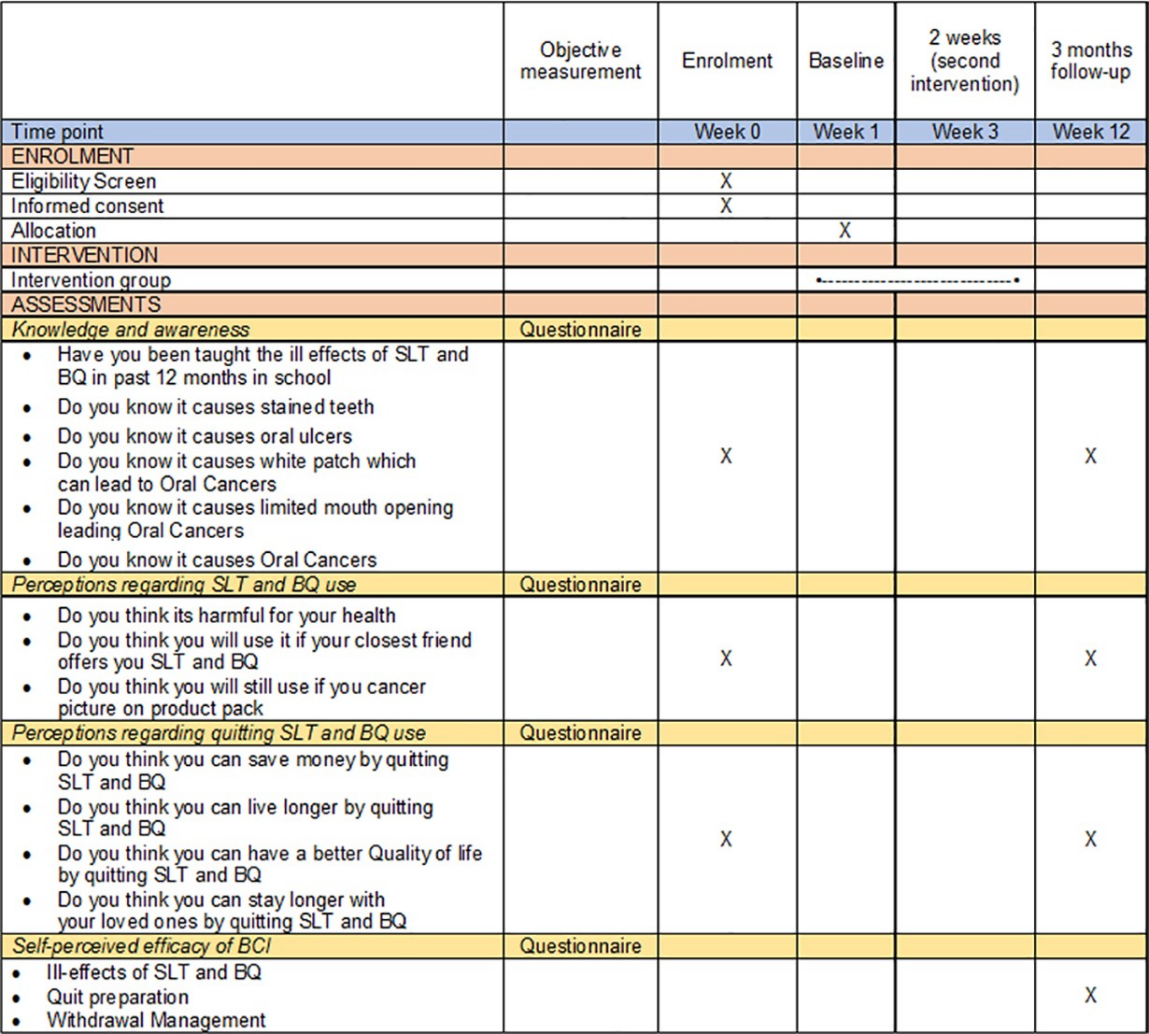
SPIRIT (Standard protocol items: Recommendations for interventional trials)[30].

All participants were given a gift hamper comprising a branded tooth paste (identity hidden to avoid conflict of interest), a tooth brush (identity hidden to avoid conflict of interest) and a pencil with imprints “SAY NO TO CHALIA (betel nut) AND GHUTKA (a form of SLT)”. The participants were also given a quit calendar to keep a record of their quit attempts that they brought back after 12 weeks (Fig 2).

The participants of the control group filled out questionnaires and were given gift hampers, educational pamphlets, and quit calendars. These participants differed the from intervention group only by virtue of the absence of the BCI.

### Questionnaire/Study variables

All students were finally selected and completed a questionnaire (modified from GYTS[31]) at the baseline and post intervention sessions. The questionnaire included demographic information (e.g., age, gender, grade in which they were studying), past and present SLT and/or BQ use history, past smoking history of any form, family history, perceptions regarding the adverse health effects of SLT and/or BQ use, motivation to quit, and perception regarding warning labels if pasted on SLT and BQ packets in Pakistan). The questionnaire was translated into Urdu (local language) and then back into English to maintain uniformity and harmony.

#### Participants’ baseline characteristics (both intervention and control arms)

A descriptive analysis was performed for the baseline characteristics regarding demographic information (e.g., age, gender, grade in which they are studying), past and present SLT and BQ use history, and type and frequency of chews.

#### Knowledge regarding the ill effects of SLT and/or BQ use among adolescents (both intervention and control arms)

There were six items that were related to the knowledge among adolescents regarding the harmful effects of SLT and BQ use in the oral cavity. All harmful consequences were scored (No = 0 and Yes = 1) depending on the participants’ awareness about them. A composite knowledge score was then generated, with a minimum score of 0 and a maximum score of 6.

#### Perceptions regarding SLT and/or BQ use among adolescents (both intervention and control arms)

There were three items related to the behaviors among adolescents regarding the use of SLT and BQ. All the perceptions were scored (No = 0 and Yes = 1) depending on the participants’ responses to them. One item was based on their perception regarding a warning label on SLT and BQ product packs (No = 0 and Yes = 1). A composite perception use score was then created, with a minimum score of 0 and a maximum score of 3.

#### Perceptions regarding cessation of SLT and/or BQ use among adolescents (both intervention and control arms)

There were four items related to the behaviors among adolescents regarding the cessation of SLT and BQ use. All cessation-related perceptions were scored (No = 0 and Yes = 1) depending on the participants’ perceptions about them. The cessation-related composite perception score (0–4) was then produced.

#### Components of the BCI that assisted participants in changing their perceptions and/or cessation

The BCI was divided into three components: product identification and the ill-effects of SLT & BQ, quitting preparation, and withdrawal management. Each component was scored as 1 = helpful and 2 = not helpful, depending on the participants’ responses (Fig 2).

### Statistical analysis

Data concerning each behavior was collected after 12 weeks in both the intervention and control groups. First, behaviors regarding knowledge and perceptions were assessed using binary responses (No = 0 and Yes = 1), which were then reported by their frequency and percentages. Further, a composite score was generated for each knowledge- and behavior-related item by summing all the responses, and descriptive statistics were reported by their means and standard deviations. The self-perceived efficacy of the BCI components was also assessed by performing the chi-squared analysis. Cohen’s d effect sizes were calculated to measure the effects of the BCI in changing the perceptions related to SLT and BQ use in both the intervention and control arms. Generalized estimating equation (GEE) models were used to determine the effects of the BCI on individual behaviors using the post-behavior scores after controlling for the baseline behavior scores. Time (pre and post) and condition (control and intervention) variables were chosen as factors, and all other covariates (age, gender, weekly pocket money and school type) were adjusted for their confounding effect. The GEE was also repeated to detect a change in the knowledge and perception scores after stratifying the data on users and nonusers of SLT and BQ. The results were reported as adjusted beta coefficients with 95% confidence intervals and p values. A p-value <0.05 was considered significant, and all analyses were performed using SPSS version 21 and STATA version 14.

## Results

In total, 2200 students from 26 schools participated in the intervention. The missing information from 60 participants decreased the sample to 2140, which was what the final analyses were run on. The intervention group comprised 1185 individuals, whereas 955 students composed the control group at baseline. After the intervention, 169 respondents (combined intervention and control groups) were lost to follow-up due to their absence on the day of our school visit; thus, the retention rate was 92% (data shown elsewhere).

In the control arm, 40% of participants were chewers of SLT and BQ, and this was reduced to 25% at follow-up, with an 8% quit rate, while, in the intervention arm, the starting rate was 44.7% and was reduced to 11.6%. In the intervention arm, 29% of the users ceased their use after the intervention.

Overall, the knowledge and awareness regarding harmful effects of SLT and BQ on oral health improved/increased between groups. The knowledge related to the transformation of oral white patch/lesion into oral cancer increased from 78% to 97.2% in intervention group, whereas it improved from 80.3% to 96.9% in control group. The awareness associated with SLT and BQ that they can cause oral cancer was already known, but it further improved in both groups.

The opinions of adolescents that were pertinent to SLT and BQ use were determined with three items. The perception regarding its harmful effect on their health changed/improved to 91.3% from 78.3% in the intervention group, while it marginally increased to 76.6% from 75.7% in the control group. However, the incorrect perception regarding the initiation of SLT and BQ was reduced to 10.0% from 20.3% in the intervention group vs. the control group (16.7% from 22.3%).

The perceptions of students related to the cessation SLT and BQ were assessed by four items; they all improved in both the study groups (Table 1).

**Table 1:** Characteristic behaviors of study groups.

				CONTROL Pre—955 Post—869		INTERVENTION Pre—1185 Post– 1102	
Characteristic Behaviors							
				Yes	No	Yes	No
Regarding SLT and BQ use				n (%)	n (%)	n (%)	n (%)
1	SLT and BQ use						
			Pre	382 (40.0)	573 (60.0)	530 (44.7)	655 (55.7)
			Post	218 (25.0)	581 (66.8)	128 (11.6)	661 (59.9)
			Quit	70 (8.0)		321 (29.0)	
Knowledge and Awareness							
1	Have the ill effects of SLT and BQ been taught in school in the past 12 months						
			Pre	755 (79.1)	200 (20.9)	912 (77.0)	273 (23.0)
			Post	743 (85.5)	126 (14.5)	1003 (91.0)	99 (9.0)
2	Do you know it causes stained teeth						
			Pre	903 (94.6)	52 (5.4)	1088 (91.8)	97 (8.2)
			Post	854 (98.3)	15 (1.7)	1086 (98.5)	16 (1.5)
3	Do you know it causes oral ulcers						
			Pre	854 (89.4)	101 (10.6)	1064 (89.8)	121 (10.2)
			Post	833 (95.9)	36 (4.1)	1088 (98.7)	14 (1.3)
4	Do you know it causes white patches, which can lead to oral cancers						
			Pre	767 (80.3)	188 (19.7)	924 (78.0)	261 (22.0)
			Post	842 (96.9)	27 (3.1)	1071 (97.2)	31 (2.8)
5	Do you know it causes limited mouth opening, leading to oral cancers						
			Pre	815 (85.3)	140 (14.7)	1004 (84.7)	181 (15.3)
			Post	828 (95.3)	41 (4.7)	1065 (96.6)	37 (3.4)
6	Do you know it causes oral cancers						
			Pre	906 (94.9)	49 (5.1)	1105 (93.2)	80 (6.8)
			Post	858 (98.7)	11 (1.3)	1086 (98.5)	16 (1.5)
Perceptions regarding SLT and BQ use							
1	Do you think it’s harmful for your health						
			Pre	723 (75.7)	232 (24.3)	928 (78.3)	257 (21.7)
			Post	666 (76.6)	203 (23.4)	1006 (91.3)	96 (8.7)
2	Do you think you will use SLT and BQ if your closest friend offers it to you						
			Pre	213 (22.3)	742 (77.7)	240 (20.3)	945 (79.7)
			Post	145 (16.7)	724 (83.3)	110 (10.0)	992 (90.0)
3	Do you think you will still use it if you see pictures of cancer on product packs						
			Pre	80 (8.4)	875 (91.6)	77 (6.5)	1108 (93.5)
			Post	22 (2.5)	847 (97.5)	30 (2.7)	1072 (97.3)
Perceptions regarding SLT and BQ Quitting							
1	Do you think you can save money by quitting SLT and BQ						
			Pre	842 (88.2)	113 (11.8)	1004 (84.7)	181 (15.3)
			Post	819 (94.2)	50 (5.8)	1033 (93.7)	69 (6.3)
2	Do you think you can live longer by quitting SLT and BQ						
			Pre	883 (92.5)	72 (7.5)	1100 (92.8)	85 (7.2)
			Post	828 (95.3)	41 (4.7)	1064 (96.6)	38 (3.4)
3	Do you think you can have a better QoL by quitting SLT and BQ						
			Pre	874 (91.5)	81 (8.5)	1106 (93.3)	79 (6.7)
			Post	827 (95.2)	42 (4.8)	1077 (97.7)	25 (2.3)
4	Do you think you can stay longer with your loved ones by quitting						
			Pre	910 (95.3)	45 (4.7)	1127 (95.1)	58 (4.9)
			Post	842 (96.9)	27 (3.1)	1092 (99.1)	10 (0.9)

BCI—Behavior Changing Intervention

Pre—First time

Post- After 3 months

QoL- Quality of life

### Univariate analysis

The part of the BCI that focused on product identification and the hazardous effects of SLT and BQ significantly helped users, nonusers and quitters of the product (p-value 0.024) in changing their perceptions (of both users and nonusers) and in quitting (in users). More interestingly, the cessation preparation module of the BCI convincingly and significantly helped the participants to attain the pertinent objectives (p-value 0.005). However, withdrawal management remained insignificant (p-value 0.383) in playing any role in the efficacy of BCI as reported by both users and nonusers of the intervention arm of the trial (Table 2).

**Table 2:** Assessing the self-perceived efficacy of the behavior changing intervention by users and nonusers of the intervention arm.

BCI components: The components that helped participants change their perceptions regarding SLT & BQ use and cessation				Nonusers	Users	Quitters	P-value
				N/%	N/%	N/%	
1	Identify the products and ill effects of SLT and BQ						
			Helpful	580 (99.3)	116 (96.7)	395 (99.2)	0.024
			Not helpful	4 (0.7)	126 (3.3)	3 (0.8)	
2	Quit preparation						
			Helpful	570 (97.6)	110 (91.7)	384 (96.5)	0.005
			Not helpful	14 (2.4)	10 (8.3)	14 (3.5)	
3	Withdrawal Management						
			Helpful	525 (89.9)	103 (85.8)	351 (88.2)	0.383
			Not helpful	59 (10.1)	17 (14.2)	47 (11.8)	

The mean knowledge score changed from the baseline score of 5.23 (95% CI 5.15–5.30) to 5.70 (95% CI 5.65–5.74) after 12 weeks of follow-up for the control arm, while the score improved from a baseline score of 5.14 (95% CI 5.06–5.21) to 5.79 (95% CI 5.75–5.82) in the intervention group during the same follow-up period.

At baseline, the control vs. intervention mean perception scores for the use of SLT and BQ were 2.45 (95% CI 2.40–2.49) vs. 2.51 (95% CI 2.47–2.54). However, after the intervention, these scores were 2.57 (95% CI 2.53–2.60) and 2.78 (95% CI 2.75–2.80) in the control and intervention groups, respectively.

The average baseline perception score for the cessation of SLT and BQ in the control arm was 3.67 [(95% CI 3.62–3.71), and it changed to 3.81 (95% CI 3.77–3.84)]; in the intervention group, this score started at 3.65 [(95% CI 3.60–3.69) and improved to 3.87 (95% CI 3.84–3.89)] after the intervention (Table 3).

**Table 3:** The effect of behavioral intervention regarding SLT and BQ use perceptions: Pre and post observation using GEE.

		CONTROL	INTERVENTION	β (95% CI)	p-value
Characteristic Behaviors					
		Mean (95% CI)	Mean (95% CI)		
**Knowledge score**					
	Pre	5.23 (5.15,5.30)	5.14 (5.06,5.21)	(a) Ref.	
	Post	5.70 (5.65,5.74)	5.79 (5.75,5.82)	0.476 (0.410,0.543)	<0.001
	**Interaction (TimexCondition)**				
		InterventionxPost		0.176 (0.078,0.274)	<0.001
	**Cohen's d (95% CI)**	0.46 (0.37,0.56)	0.63 (0.55,0.72)		
**Perception scores regarding use**					
	Pre	2.45 (2.40,2.49)	2.51 (2.47,2.54)	(b) Ref.	
	Post	2.57 (2.53,2.60)	2.78 (2.75,2.80)	0.132 (0.096,0.168)	<0.001
	**Interaction (TimexCondition)**				
		InterventionxPost		0.141 (0.090,0.192)	<0.001
	**Cohen's d (95% CI)**	0.19 (0.10,0.28)	0.47 (0.39,0.56)		
**Perception scores regarding quitting**					
	Pre	3.67 (3.62,3.71)	3.65 (3.60,3.69)	(c) Ref.	
	Post	3.81 (3.77,3.84)	3.87 (3.84,3.89)	0.143 (0.102,0.185)	<0.001
	**Interaction (TimexCondition)**				
		InterventionxPost		0.067 (0.006,0.129)	0.031
	**Cohen's d (95% CI)**	0.21 (0.12,0.30)	0.33 (0.24,0.41)		

SLT: smokeless tobacco, BQ: betel quid.

GEE: generalized estimating equation, β: Beta coefficient, CI: confidence intervals.

(a): Adjusted model for knowledge score regarding the ill effects of SLT and/or BQ with the following variables: age, gender, school type and weekly pocket money.

(b): Adjusted model for perception score regarding SLT and/or BQ use with the following variables: age, gender, school type and weekly pocket money.

(c): Adjusted model for perception score regarding quitting the use of SLT and/or BQ with the following variables: age, gender, school type and weekly pocket money.

Interventionxpost–after 12 weeks in intervention group.

Timexcondition—time–post 12 week of intervention and condition–intervention group.

#### Change observed in knowledge, perception and quit scores at 12 weeks of follow-up

Overall, we observed an increase in knowledge scores of 0.476 units (95% CI 0.410–0.543, p-value <0.001) after adjusting for the covariates of age, gender, school type and weekly pocket money after the follow-up; furthermore, a 0.176-unit (p-value <0.001) increase in the knowledge scores was found in the intervention group after the BCI compared with that of the control group. The Cohen’s d of 0.63 (95% CI 0.55–0.72) suggested a large positive effect of BCI in changing the participant knowledge base related to SLT and BQ use in the intervention arm compared with that of the control arm 0.46 (95% CI 0.37–0.56).

The perception score regarding the use of SLT and BQ after BCI was noted to be increased by 0.132 units (95% CI 0.096–0.168, p-value <0.001), while a 0.141-unit (p-value <0.001) increase was found in the intervention group after the BCI compared with that of the control group. A moderately positive effect of BCI in the intervention arm of the study was observed by a Cohen’s d of 0.47 (95% CI 0.39–0.56) compared with that of the control group [0.19 (95% CI 0.10–0.28)] that exhibited a small effect in changing the perceptions regarding SLT and BQ use.

The adjusted model that was run for the perceptions regarding SLT and BQ cessation detected a 0.143-unit (95% CI 0.102–0.185, p-value <0.001) increase after the follow-up; however, a 0.067-unit (p-value 0.031) increase was seen in the intervention group after BCI compared with that of the control group. BCI was suggested to be marginally effective in changing quit perceptions over the control group as concluded by a Cohen’s d of 0.33 (95% CI 0.24–0.41) in the intervention group versus a value of 0.21 (95% CI 0.12–0.30) in the control arm (Table 3).

#### Change observed in the knowledge and perception scores at 12 weeks of follow-up after stratification at the user & nonuser level

Stratification was done at the level of users and nonusers to identify changes in SLT and BQ related knowledge and perceptions, and significant changes were revealed in both strata. The effect sizes of the changes in knowledge scores (β: 0.651 95% CI 0.532–0.770, p-value <0.001), use perceptions (β: 0.201 95% CI 0.148–0.272, p-value <0.001) and quitting perceptions (β: 0.182 95% CI 0.113–0.250, p-value <0.001) in users were greater and highly significant than those of nonusers (Table 4).

**Table 4:** Stratified sample analysis between users and nonusers with SLT and BQ use: GEE.

Characteristic Behaviors		Nonusers		Users	
		Pre– 1228		Pre– 912	
		Post—1085		Post– 886	
		β (95% CI)	p-value	β (95% CI)	p-value
**Knowledge score (a)**					
	Pre	Ref.		Ref.	
	Post	0.362 (0.287,0.437)	<0.001	0.651 (0.532,0.770)	<0.001
Interaction (TimexCondition)					
	InterventionxPost	0.186 (0.064,0.308)	0.003	0.129 (-0.033,0.291)	0.119
Age					
	< 12 years	Ref.		Ref.	
	≥ 12 years	0.184 (0.084,0.283)	<0.001	0.136 (0.001,0.272)	0.048
Gender					
	Male	Ref.		Ref.	
	Female	-0.167 (-0.257,0.066)	0.001	-0.080 (-0.199,0.40)	0.191
Weekly pocket money					
	≤ 100	Ref.		Ref.	
	> 100	-0.178 (-0.314,0.043)	0.010	0.056 (-0.199,0.312)	0.666
School type					
	Private	Ref.		Ref.	
	Government	0.032 (-0.070,0.133)	0.540	0.224 (0.103,0.344)	<0.001
**Perception scores regarding use (b)**					
	Pre	Ref.		Ref.	
	Post	0.083 (0.040,0.126)	<0.001	0.210 (0.148,0.272)	<0.001
Interaction (TimexCondition)					
	InterventionxPost	0.111 (0.049,0.173)	<0.001	0.157 (0.074,0.241)	<0.001
Age					
	< 12 years	Ref.		Ref.	
	≥ 12 years	-0.027 (-0.078,0.023)	0.287	-0.102 (-0.179, -0.025)	0.009
Gender					
	Male	Ref.		Ref.	
	Female	-0.001(-0.055,0.054)	0.978	-0.075 (-0.154,0.005)	0.065
Weekly pocket money					
	≤ 100	Ref.		Ref.	
	> 100	-0.036 (-0.110,0.039)	0.352	-0.149 (-0.264, -0.033)	0.012
School type					
	Private	Ref.		Ref.	
	Government	-0.045 (-0.104,0.014)	0.134	-0.178 (-0261, -0.096)	<0.001
**Perception scores regarding quitting (c)**					
	Pre	Ref.		Ref.	
	Post	0.116 (0.064,0.168)	<0.001	0.182 (0.113,0.250)	<0.001
Interaction (TimexCondition)					
	InterventionxPost	0.047 (-0.027,0.121)	0.211	0.087 (-0.015,0.189)	0.094
Age					
	< 12 years	Ref.		Ref.	
	≥ 12 years	0.041 (-0.019,0.100)	0.180	0.025 (-0.055,0.105)	0.541
Gender					
	Male	Ref.		Ref.	
	Female	0.064 (0.005,0.123)	0.035	0.041 (-0.034,0.116)	0.286
Weekly pocket money					
	≤ 100	Ref.		Ref.	
	> 100	0.023 (-0.070,0.116)	0.631	0.096 (-0.067,0.260)	0.248
School type					
	Private	Ref.		Ref.	
	Government	0.058 (-0.001, -0.116)	0.053	0.100 (0.029,0.172)	0.006

(a): Adjusted model for knowledge score regarding the ill effects of SLT and/or BQ with the following variables: age, gender, school type and weekly pocket money.

(b): Adjusted model for perception score regarding SLT and/or BQ use with the following variables: age, gender, school type and weekly pocket money.

(c): Adjusted model for perception score regarding quitting the use of SLT and/or BQ with the following variables: age, gender, school type and weekly pocket money.

β: Beta coefficient.

CI: Confidence Interval.

## Discussion

The current study shows that the BCI significantly changed the knowledge and perceptions regarding SLT and BQ use and cessation in a promisingly positive direction. Although the increase in knowledge and positive perception changes were also noted in the nonintervention group, in comparison to the intervention group, they were not significant; thus, this finding further strengthened the evidence-based efficacy of the BCI.

The results of the current study also suggested that a culturally rooted, school-based behavior changing intervention was an effective way to enhance the knowledge and awareness of school-going adolescents regarding the health hazards that are pertinent to SLT and BQ use. Such school-based interventions for tobacco (both smoked and chewed) control have already shown promising results in creating awareness and preventing substance abuse [17, 32–34]

We anticipated differences in the effect sizes of BCI in government and private schools due to the differences in the socioeconomic circumstances of students in each school type, the availability of the product in different types of schools and the differing cultural backdrop of each school type. In contrast, our results were in disagreement with the previous similar interventions that had detected different effect sizes of interventions in which there was a sociodemographic inequality that existed between schools [19, 35].

The study revealed changes in the knowledge and perceptions of the users and nonusers in both study arms, which was analogous to results of past studies [18, 36] where the objectives of the intervention were achieved in both arms. Similar to the present study, these studies did not include students who had motivations to quit; therefore, the SLT quit rate of the current study (29% in the intervention group) was comparable to that of past interventions [27%[18]and 30.2%[36] in the intervention arm].

In the current study, the effect sizes of changes in knowledge and perceptions were significantly more encouraging in the users than those of the nonusers. These results were desirable since adolescents who possess more pro-use behaviors regarding SLT are more likely to use them and to become smokers in adulthood [37].

The BCI was effective in both the users and nonusers in the intervention group in this study. The intervention was convincingly helpful in changing the perceptions of the participants that the use of SLT is detrimental for oral health specifically and overall health in general. Furthermore, by quitting, participants can achieve lifelong benefits, and their quality of life can improve remarkably. These results were in accordance with those of trials in the past [18, 36], where it was concluded that such interventions do increase abstinence from SLT irrespective of users’ pre-existing motivations to quit. Furthermore, telephone counselling may also serve as an effective mode of imparting awareness and attaining cessation among users regardless of whether they are in the intervention or control arm; similar results were noticed in our study, where we sent awareness messages via telephone to the participants of the control arm, which prompted them to quit.

The reason for the ineffective behavioral programs in the past was also related to the absence of the identification of components of the BCI that might have prompted positive results [15, 16]. In the current study, the assessment of the effective components of the BCI by the participants themselves may play a role in designing future customized BCIs. In addition, the results of the self-professed efficacy of two components of BCI (the knowledge and awareness of the hazardous effects of SLT & BQ, along with the ways to tackle cessation of these products) may be extrapolated to be beneficial if they are incorporated in the school curricula (as also suggested by a meta-analysis of 50 randomized control trials of school-based smoking prevention curricula[38]). Similarly, it was concluded that children may remain never-smokers when social influence is incorporated into the interventions that are part of the curricula. Similar comparable results of interventions that suggested the incorporation of intervention in integrated primary care to ensure the cessation of smoking were also found [39].

To the best of our knowledge, this is the first study in Pakistan where a large high-risk group of school-going adolescents was intervened with a BCI that was intertwined with the cultural fabric of use of those products, which are otherwise identified as acceptable to consume.

We were able to connect with high-risk adolescents in schools and were able to inform them about the health hazards of SLT and BQ, either with the help of the BCI or by educational pamphlets that were distributed in the control arm, thereby imparting health awareness to these adolescents.

Schools were also informed about the positive results of the study, and suggestions were made to make these awareness sessions part of their curricula.

This study had some limitations. First, this study was based on adolescents in urban schools; thus, the results may not be generalized to the same group in smaller rural areas. Second, students might not have reported their knowledge and perception changes with accuracy since these questions could be misinterpreted; however, the reliability and validity of the questionnaire that we used has been indicated in the literature as having good test-retest and good fit of the results [40–43]. Lastly, the results did not measure the long-term efficacy of the BCI; thus, RCTs of a longer duration may elicit more pronounced and robust results of such programs.

The results of the current study robustly demonstrate the efficacy of school-based interventions to curb the chewing habit of SLT and BQ in adolescents. Such interventions may play a positive role in improving students’ knowledge base and inculcate positive healthy perceptions regarding SLT and BQ use, thereby promoting abstinence from these risky substances. It may also be suggested to make such interventions part of school curricula to keep these schools SLT and BQ use-free.

## Conclusion

The usefulness of BCI in improving adolescents’ knowledge and positively changing their perceptions regarding harmful SLT and BQ use can safely be concluded from the current study. Additionally, convincing results may be achieved if interventions are tailored, with more emphasis on the identification of the products that are used by the adolescents in addition to highlighting their ill-effects and how students can manage to quit by overcoming cessation-related hurdles. Such BCI, if included in schools’ curricula, may create “SLT and BQ use-free schools”.

## Supporting information

S1 Checklist CONSORT 2010 checklist. (DOC)

S1 File BCI protocol and appendices plos one. (PDF)

S1 Dataset De-identified data. (SAV)
