# Supplementary material for: School-based behavioral intervention to reduce the habit of smokeless tobacco and betel quid use in high-risk youth in Karachi: A randomized controlled trial
Source: PLoS One. 2018 Nov 2;13(11):e0206919. doi: 10.1371/journal.pone.0206919 (PMC6214566; doi:10.1371/journal.pone.0206919)
Supplement: S1 File — (PDF) [file pone.0206919.s002.pdf]

Behavior Changing Intervention for smokeless tobacco and betel quid use in adolescents: A cluster-randomized trial

A Thesis proposal submitted to (Institute of Basic Medical Sciences), DUHS, in the partial fulfillment of the requirement for the Program of

Doctor of Philosophy

(Public Health)

Institute of Basic Medical Sciences

Dow University of Health Sciences

February 14, 2016

Revised version: April 04, 2016

Approved by:

____

Prof. Masood Qureshi

Director, IBMS

DUHS

Summary of Administrative Information

NAME OF THE RESEARCHER:

Prof. Dr. Azmina Hussain

Designation:

Professor

Qualifications:

BDS, M.Phil

Signature:

SUPERVISOR:

Dr. Kashif Shafique

Designation:

Associate Professor and Vice Dean, School of Public Health

Qualifications:

MBBS (Dow), MPH (Glasgow), PhD (Glasgow)

Signature:

Contents

1 Abstract 4
1.1 Background 4
1.2 Methods 4
1.3 Key words 5
2 Introduction 6
2.1 Significance of the problem 6
2.2 Statement of the problem 7
2.3 Objectives 7
2.4 Operational definitions 8
3 Literature Review 8
4 Methodology 10
4.1 Sample description & inclusion/exclusion criteria 10
4.2 Research Design 10
4.3 Sample Size Calculation 10
4.4 Informed consent and Recruitment 10
4.5 Questionnaire (appendices 1 & 2) – Baseline measures 11
4.6 Formulating the Behavior Changing Intervention (appendix 4) 11
4.6.1 The components of BCI include: 12
4.7 Intervention and Control Groups (Randomization and concealment) 12
4.8 Accuracy of intervention delivery (appendix 3) 12
4.9 Quit measures 13
4.10 Oral examinations 13
4.11 Follow up after four weeks 13
4.12 Plan of Analysis 14
4.12.1 Participants' baseline characteristics (both intervention and control arms) 14
4.12.2 SLT/or BQ consumption(47) – users of both intervention and control arms 14
4.12.3 Reasons for Betel quid chewing scale(47) – users of both intervention and control arms 14
4.12.4 Betel Quid Dependence Scale (BQDS)(49) – users of both intervention and control arms 14
4.12.5 Fagerstörm Tobacco and Nicotine Dependency Scale (FTND-ST)(48) – users of both intervention and control arms 14
4.12.6 Perceptions regarding ill effects of SLT/or BQ use among adolescents (intervention arm) 14
4.12.7 Quit measures 14
4.12.8 Oral examinations 14
4.13 Ethical considerations 15
5 Study Time line & Schedule 15
6 Appendices 16
7 References 17

1 Abstract

1.1 Background

Smokeless tobacco (SLT) and Betel Quid (BQ) are known risk factors for Oral, Pharyngeal and Esophageal carcinoma. Three-quarters global SLT consumption is among the South Asian Population. The habit of SLT and BQ chewing commences at a very young age which has an underpinning of socio-cultural dimension in South Asian population which perhaps due to its more addictive potential, has more dependency. Youth also perceives SLT and BQ as a part of confectionery, which is socially served in South Asian ceremonies. There exists very little or no evidence regarding efficacy of SLT and BQ cessation interventions in this population. Most of the existing interventions are based in western world with little or no cultural sensitivities pertinent to South Asia. We developed a culturally rooted behavior changing intervention (BCI) to alter SLT use prevalence, perceptions pertinent to the deleterious effects of SLT and BQ use in the etiology of oral cancers and help youth quit SLT and BQ in Karachi, Pakistan.

1.2 Methods

Baseline demography and SLT & BQ use prevalence will be ascertained among 11-16-year-old school going children from both government and private schools. Cluster sampling will randomly recruit 26 clusters (schools) from within 6 districts of Karachi, which will then be divided into intervention and control groups (clusters) using block randomization based on proportionate number of each school type present (Government and Private).

Both groups' participants will complete all questionnaires pre and post intervention as described elsewhere and will also undergo screening for oral cancer and oral potentially malignant lesions (OPMLs). Students in intervention cluster will be given BCI and printed pamphlets along with a gift pack (reminder for SLT and BQ quit, a branded tooth paste and a tooth brush) while students in control cluster will only differ in that they will not be exposed to BCI.

BCI was designed after reviewing literature and consulting specialist group for all untoward effects of SLT and BQ use in Oral Cavity that have a potential of transforming into oral carcinoma. Identification of all determinants of SLT and BQ use was fundamental for the identification of behavior changing techniques (BCTs) which can possibly modify the behavior of SLT & BQ users and help youth in quitting. The BCTs were also sensitively incorporated into BCI by keeping South Asian culture as a backdrop. The accuracy of BCI will be assessed by fidelity index modified to score adherence and quality of the components of BCI.

A follow up after 12 weeks will be conducted to re-assess their SLT and BQ use prevalence, perceptions regarding hazardous effects of SLT & BQ use in oral cavity, dependency on SLT & BQ and success in quit (among users in both groups), and perception regarding warning labels on SLT & BQ product packet.

1.3 Key words

Tobacco, smokeless, chewing, betel quid, South Asian, behavioral support, behavior change, behavior changing technique, fidelity index, cessation, Oral cancer, adolescents, screening

2 Introduction

2.1 Significance of the problem

Smokeless tobacco(SLT) is a widely used term that includes varying types of tobacco products that are used orally or put in nose(1). It includes betel quid (contains areca nut with or without tobacco), ghutka, naswar, zarda, mawa, snuff (both moist and dry), chewing tobacco and many other dissolvable tobacco products(2). SLT is consumed worldwide in many forms which may include unprocessed that is dried in sunlight or processed products, that are either chewed, snuffed, sucked or placed in the oral cavity between lips and buccal mucosa(3).

In approximately 115 countries, more than 300 million adults consume SLT in various forms(4); the majority of the consumers (89%) are concentrated in South Asian countries(5). In South Asian Countries, SLT use prevalence is more in males than females; less than one third of female population consume SLT in Mauritania (28.3%), and India (18.4%) whereas, more than one third of male population of Myanmar (51.4%), and India (32.9%) consume SLT. Within Europe, SLT in the form of Snus is more commonly consumed with similar trend of higher consumption in males (24% and 20% in Sweden and Norway respectively) as compared to females which is 7% and 6% in Sweden and Norway respectively (4). In Pakistan, the regular consumption of SLT is 16.3% and 2.4% amongst males and females respectively (6). In school going adolescents; according to a study based on GYTS (global youth tobacco survey)(7), 18.5% boys and 8.4% girls chew SLT that is similar to the more male and less female consumption trend as in adults. According to another study in India, within 10 % SLT use prevalence 80.9% users were males(8). In Karachi, school going adolescents of 13-15 years; according to GYTS-2008, 16.7% boys and 9.6% girls consume SLT(9).

SLT consumption is accepted in South Asian countries as part of their cultural and social norms(10). At a very young age, individuals start consuming it as they are readily available and considered as sweets(11). A variety of factors and list is not exhaustive to family values, easy access, cheap in cost, as dental pain relieving medicine, no enforcement of legislation on its use, are responsible for SLT wide spread use(12, 13). The women and young individuals in India, Pakistan and Bangladesh refrain from smoking due to cultural and traditional beliefs but there is no such hesitation in SLT use; rather parents encourage their children to use it(14).

Considerably variable modes of marketing like in Indian cinema, celebrities use these ghutka products as something very “cool” and then it is sold in very attractive packaging. The youth considers it as very popular, acceptable product and subsequently becomes addictive to it(15). School going children also get fascinated by the undesirable marketing of SLT products and spend the substantial amount of their pocket money on the purchase of these dangerous substances(8, 16). Majority of the users get engaged in this habit even before 15 years of age due to influence by media (40.2%) and family & friends pressure (30.8%)(17) while in another study, peer pressure (76%) was reported(8). In a study by Khan A. (18); in India, Pakistan, Nepal and Bangladesh, there are either no policies to control sell and purchase of SLT or they are very inadequate. Once adolescents start consuming SLT, it becomes an essential component of their lives since SLT is the fourth commonly abused substances used worldwide and develop dependency after alcohol, nicotine & caffeine with both stimulating and relaxing effect(19-21).

2.2 Statement of the problem

SLT contains nicotine and carcinogenic nitrosamines which cause more than a quarter million deaths mainly due to oral and pharyngeal cancers(4). Betel Quid(pan) with or without tobacco, areca nut (supari, chalia), ghutka, niswar, tobacco smoking in all forms are proven oral carcinogens by IARC (International Agency for Research on Cancer)(22). Areca nut alone, in formulation as betel quid and other SLT chewable are proven oral cavity carcinogens(19, 22) and are chewed by 600 million people worldwide specially in South Asia(23). SLT is also a risk factor for oral potentially malignant lesions like oral sub mucus fibrosis, leukoplakia, dysplastic mucosal changes, and oral ulcers(3).

Oral cancer incidences are increasing with decreasing survival(24) rates to approximately below 50% despite of extensive treatments given at tertiary care hospitals. The World Health Organization (WHO) reports that the trend of such cancers would increase further in South Asian countries where incidences of oral cancers are more as compared to rest of the world(25). In developed world, alcohol and tobacco are established risk factors of oral cavity tumors(26). According to a systematic review regarding SLT use and oral cancer in South Asia(27), there is a very profound association between SLT consumption and oral cancer where betel quid with tobacco chewers have seven times more risk of developing oral cancer than non-chewers. Also, the risk of oral cancer in SLT chewers other than betel quid is five times more than in non-chewers(27). In Pakistan, according to Shaukat Khanum cancer registry, in both genders above 18 years of age; lip and oral cavity carcinoma is third most carcinoma(28). Areca nut, betel quid, and tobacco increase the risk of oral cancer by 8.5-10 times as per reported in a study in Pakistan(10).

2.3 Objectives

In lieu of the increasing disease burden of oral cancer in South Asian countries due to increasing popularly fashionable SLT consumption; our objective is to develop (i) a BCI pertinent to socio-cultural aspect for South Asian SLT users, followed by (ii) assessing its efficacy in changing perception and dependency regarding SLT use in high risk group of 11-16 years old school going children of Karachi of both private and public sector schools.

We also aim to deconstruct our BCI and establish which components of our BCT were more useful in making an intervention most efficient by using sub-indices of fidelity index to assess the adherence and quality of each component of intervention. Each component will be scored using fidelity index by a neutral trained listener each time intervention is delivered by a trained personnel.

Quantitatively, our objectives of the study are to:

i. measure SLT and BQ use prevalence amongst adolescents of 11-16 years
ii. assess the reasons behind their use of SLT and BQ in users (both intervention and control clusters) by using Reasons for Betel Quid Chewing Scale (RBCS).
iii. evaluate the dependency on SLT and BQ in users (both intervention and control clusters) using Fagerstörm Tobacco and Nicotine Dependency scale for Smokeless Tobacco (FTND-ST) and Betel Quid Dependency by using Betel Quid Dependency Scale (BQDS).
iv. assess perceptions of adolescents (both intervention and control clusters) regarding ill effects of SLT and BQ use in oral cavity causing oral cancer and OPMLs.
v. assess the perception of adolescents regarding oral cancer picture of SLT and BQ product pack in cessation of its use with the help of Behavior Changing Intervention in intervention cluster and a questionnaire item in control cluster.

vi. evaluate each component of Behavior Changing Intervention in its efficacy by using fidelity index and self-perceived efficacy by participants of intervention cluster.

2.4 Operational definitions

- Smokeless tobacco (SLT): It includes

i. betel quid with tobacco,
ii. ghutka (betel leaf, areca nut and lime),
iii. naswar,
iv. zarda,
v. mawa,
vi. snuff (moist ground tobacco),
vii. chewing tobacco,
viii. snus (finely ground moist tobacco) and
ix. many other dissolvable tobacco products that are put in mouth or nose

- Betel Quid (BQ): A ‘betel quid’ (synonymous with ‘pan’or ‘paan’) generally contains betel leaf, areca nut and slaked lime
- Areca nut alone, without any betel leaf, slaked lime or tobacco is another variety that is chewed.
- Behavior changing techniques (BCTs) are defined as “any explicit description of intervention content that can alter a participant’s “chewing SLT/or BQ behavior”
- Behavior changing technique codes (BCT-Cs): BCT code is based on a function that the BCT performs

i. B - Specific focus on behavior
ii. A - Promote adjuvant activities
iii. R - General aspects of the interaction
iv. M - addressing motivation
v. S - maximizing self-regulatory capacity/skills
vi. D - focusing on delivery of the intervention
vii. I – gathering information
viii. C - focusing on general communication

- **Current SLT and BQ use: “Current smokeless tobacco and betel quid use is defined as ever using such products and using an smokeless tobacco/betel quid product at least once within the past 30 days”(29).**

3 Literature Review

Individuals at a very young age start consuming SLT with a mean age of initiation being 13 years (SD 7.07) which earlier was 15 years, suggestive of an early initiation of SLT in this era(8). Key factors that played role in initiation

also play role in quitting decision along with dentists' advice(13). But there is also no adequate support available for people who wish to quit(18). Thus, no support for quit and loneliness lead to relapse(30). The effectiveness of behavioral interventions in helping SLT users in quitting was greatly supported by a Cochrane review but its effect size was limited since there was ambiguity in which part of the intervention helped the most to have its impact(31). It was also concluded in the review that pharmacological interventions like varenicline and nicotinic lozenges may also support quitters in abrupt cessation(31). SLT cessation was effective in one intervention where success was attributed to follow up session along with oral examination(32), this being coherent with a Cochrane review(31) for intervention for SLT use cessation in 2011. Gansky reported dubious effects of BCI where SLT cessation effects were more pronounced in control group and reasons assigned were mostly rooted in strong legislation against SLT use in the western world and athletes who participated in this study, are more concerned about healthy living (33). Walsh *et al.* (34) randomized 44 schools either to an intervention or control group and suggested that BCI was effective in both users and non-users because SLT users were at an early stage of habit building along with less addiction as products that individuals in western countries use are less addictive. Also, their quit had an underpinning of western cultural.

A Cochrane review updated in 2015 on interventions for SLT use cessation(35) convincingly suggests, as their conclusion from 2011 review regarding behavior intervention remains unchanged; and reports from 17 behavioral interventions that the confidence in effect size of interventions was limited since which constituents of intervention influenced their impact are not clear. Alongside, in the same(35) it was updated that effect of oral screen in the success of the intervention was not observed.

Since most of the interventions (inclusive of Cochrane review(35) on interventions for SLT use cessation; updated till 2015) were designed and carried on in the western world thus the extrapolation of their effectiveness in South Asian countries was unrealistic to pre-empt(36).

Also, the rationale of incorporating more social and cultural components in BCI in South Asian countries is more justified. Most of the trials designed for SLT cessation are adapted by already conducted trials based on smoking cessation that are insensitive of socio-cultural impact of SLT use(37). A systematic review and meta-analysis strongly suggests that since SLT use has a very strong cultural backdrop attached in South Asian countires and along with scanty awareness regarding deletirious effects on oral health and illiteracy; they are bound to play a signifcant role in BCI to control SLT use amongst high risk group of 13-15 years old(38). Also, since increasing oral cancer trend in south asian countries is because of SLT which is completely avoidable thus it can serve as a lucrative initiating point to control for oral cancer and plan primary preventive interventions(38).

Identifying the gap in the existing literature, a reliable nomenclature of BCTs was developed by Michie S. et.al., in order to assess the efficacy of each component of BCI so as to augment its use in cessation interventions(39). A nomenclature of such BCTs used and their proven efficacy was recently published by Michie S. et.al(40).

4 Methodology

4.1 Sample description & inclusion/exclusion criteria

This study will be conducted at government and private schools of Karachi as schools have the maximum probability of engaging focused age group of mixed ethnicity, religion, different cultures and different socio-economic strata of the population. Also, these young individuals spend a substantial number of their waking hours in school thus they are easily approachable globally without needing to delineate or be reliant on families(41).

With a population of approximately 16 million, Karachi is the largest city of Pakistan(42). Karachi City government has divided the city into six (6) districts in 2013 with each district being further divided into administrative towns of total 18 in number(43).

Cluster sampling will be done. Out of six districts, twenty six clusters (secondary schools) will be randomly selected proportionate to the number of each school type (ensuring equal participation of government and private schools). The students of 11-16 years of age, studying in grade VI – Grade X will be randomly recruited. Complete lists of all private and government schools of Karachi have been arranged by requesting respective Directors (Appendix 6). If any randomly selected school declines to participate than another school will be randomly selected from the same district; also, a government school will be selected if government school will decline and vice versa. Fifty to 100 students per school will be randomly included in the study. Depending upon the size of each class, students will be randomly selected from each of the secondary classes present on the day of our visit till a total number of 50-100 is reached.

This will therefore summit a total sample size of 2200 that may be considered representative of the 11-16 year adolescents of Karachi as Global Youth Tobacco Survey conducted in Karachi, Pakistan in 2008 enrolled 720 individuals of 13-15 years for comprehensive tobacco control program(44).

Students of younger or older age group than mentioned already will be excluded and students who already are undergoing treatments for Oral Cancer will be excluded.

4.2 Research Design

This study is a “Cluster-Randomized Trial”.

4.3 Sample Size Calculation

Sample size calculation was based on efficacy of BCI for SLT and BQ use cessation. In openEpi; using two sided significance level at (1-alpha) 95%, an intra-cluster correlation coefficient of 0.05 (to cater for within school influences on cessation) with 90% power to detect a change of minimum 7% over a control cluster for BCI efficacy in intervention arm; 1606 sample size was calculated(9). Adding approx. 25% attrition rate, it was upsized to 2200 participants. Due to inaccessibility to software for cluster randomized trial to us, cluster size and number of clusters were calculated manually using equations which turned out to be 13 clusters in each arm with 50-100 participants in each cluster(45).

4.4 Informed consent and Recruitment

For recruitment purposes, we will contact principals of selected schools (both government and private) to provide them with details regarding the intervention, its merits and efficacy, and will request for their inclination

to participate in the trial. If any selected school refuses to participate then another school of same profile will be sent an invitation.

Schools' heads will then be requested to send consent forms (will be provide to schools) to the parents along with a covering letter in which they will be provided with all relevant details regarding intervention and will be encouraged to contact principal investigator via a text on given contact number who will then call parents to respond to all queries since we do not have toll-free numbers.

Parents will be requested to sign an acceptance or refusal on the form and sent it back to school by a specific decided date.

4.5 Questionnaire (appendices 1 & 2) – Baseline measures

All students finally selected will complete a questionnaire (adapted from GYTS(44)) at the baseline session (appendix 1). The questionnaire includes demographic information (e.g. age, gender, grade in which they are studying), past and present SLT and BQ use history, past smoking history in any form, family history, perceptions regarding adverse health effects of SLT and BQ use, motivation to quit, perception regarding warning labels if pasted on SLT and BQ packets in Pakistan). SLT and BQ users will then be identified to complete questionnaires on reasons to chew betel quid(46) to assess their rationale behind this habit, to assess nicotine dependency using Fagerstörm Tobacco and Nicotine Dependency scale for Smokeless Tobacco (FTND-ST)(47) and Betel Quid Dependency by using Betel Quid Dependency Scale(48) (Appendix 2). All the questionnaires are translated into Urdu (local language) and then back into English so as to have uniformity and harmony.

4.6 Formulating the Behavior Changing Intervention (appendix 4)

The behavior change intervention (BCI) for SLT and Betel Quid (with or without tobacco) cessation is based on changing perceptions of 11-16-year-old school going children regarding effects of SLT & BQ and to assist them in quitting this life threating habit. The methodology acquired in designing, developing and assessing the accuracy of this BCI was based on Medical Research Council framework for complex interventions(49).

The BCI is formulated by using theoretical BCTs underpinning the socio-cultural norms of South Asian populations which are apt for motivating quit of SLT and BQ in young users. Following steps were followed in its development:

1. Recognition and assortment of all key contributing factors associated with SLT & BQ use in South Asian Youth and their associated BCTs,
2. Transforming all BCTs into culturally tailored appropriated behavior changing activities which was not limited to highlighting adverse and life threatening outcomes of SLT & BQ use,
3. And assessing the feasibility of its delivery in young children by a panel of experts who assessed each component of BCI intervention based on their expertise and termed it as appropriate or inappropriate; only appropriate ones are the part of the intervention.

Since this BCI is first of its kind at such a mega level in youth of Karachi, Pakistan; a literature review was conducted to assess all untoward effects of using SLT and BQ on oral health specifically, to assess habit forming reasons of using SLT & BQ and to identify potentially malignant lesions (OPMLs)(50).

With an aim to influence youth's perception regarding discontinuing the use of these products; most recent taxonomy of BCTs was adapted to translate these key contributing factors associated with SLT & BQ use, into BCI by focusing more or oral cancer prevention /or early detection(39).

A panel of oral & maxillofacial pathologists and oral & maxillofacial surgeons based on their academic and clinical expertise, labeled each OPML as **commonly seen** in patients (in our community) with an exposure to SLT & BQ use with already having an established causal relationship and **not so commonly seen** in patients with an exposure to SLT & BQ use. Only commonly seen OMPLs were included in the BCI.

This BCI was then designed into an intervention which was culturally sound in South Asian population keeping all cultural sensitivities as a backdrop by a panel of experts who are well aware of designing such activities in South Asian communities. It was essential to be mindful of the cultural sensitivities as for south Asian individuals, it is part of their cultural norms and their parents encourage SLT use. Thus, throughout BCI, harmful effects of BCI are highlighted without stigmatizing its socio-cultural use.

A guide for behavioral support for smoking cessation(51) was kept as a guideline for designing this intervention into pre-quit, quit and post quit management of withdrawal symptoms.

4.6.1 The components of BCI include:

- Identifying the SLT and BQ products, their harmful effects, and why is it important to quit along with setting quit date
- Preparation to quit along with managing urges for relapse, and
- Recognizing and managing withdrawal symptoms

4.7 Intervention and Control Groups (Randomization and concealment)

Once schools are selected; they will be recruited into intervention and control groups by block randomization based on private or public schools so as to control for any differences in socio-economic status as the fee structure in each significantly differs (52) and wealth is directly and positively related to SLT consumption intensity for already users(53). Also, schools (clusters) will first agree to participate and then will be recruited in randomly in each intervention and control groups(33) to avoid any selection bias. However, all intervention material will be delivered to control clusters at the end of the study.

We will recruit clusters (schools) in each intervention and control group to avoid any diminution of the intervention outcome through discussion regarding intervention amongst the participants of the same school. Gansky and colleagues(33) assigned the failure of their intervention to the "spill over" effect due to the contact between athletes in intervention and control groups.

Both groups' participants will complete all questionnaires pre and post intervention as described elsewhere.

Students in the intervention group will be shown a well-structured visual presentation which will assess their perceptions regarding SLT and BQ mentioned elsewhere while controls will not be exposed to BCI. *The intervention will be delivered by trained personnel to avoid any researcher bias.*

All participants will be given a gift hamper comprising of a branded tooth paste (identity hidden to avoid conflict of interest), a tooth brush (identity hidden to avoid conflict of interest) and a pencil with imprints "SAY NO TO CHALIA AND GHUTKA". They will also be given a quit calendar to keep record of their quit attempt.

4.8 Accuracy of intervention delivery (appendix 3)

The accuracy and fidelity to the intervention will be assessed by applying two sub-indices of pre-defined fidelity index. The fidelity index is composed of (i) Adherence Scale, and (ii) quality scale. There are 20 items for adherence scale in our intervention to be assessed by fidelity index and 5 items for quality scale. Each scale has

a scoring system composed of three point Likert scale; 0=not implemented, 1=partially implemented, and 2=fully implemented(54). This is adapted from a fidelity index used in a study(55).

This index will be filled up by a trained neutral person each time BCI is delivered in small groups.

The significance of this index is to correlate intervention delivery with the outcome in the form of behavior change regarding SLT and BQ quit and identify which component worked or did not work in attaining the desired modification in the behavior. This method has an advantage of being more objective assessment of fidelity of the intervention than self-reporting(56). Participants' adherence to the intervention will be objectively assessed by the number of quit calendars that we will get back after 12 weeks. They will also help us to assess attrition and thus attrition bias if any.

4.9 Quit measures

Self-reported SLT and BQ quit will be measured by quit calendars from users of both control and intervention groups.

4.10 Oral examinations

Oral examinations will be performed using conventional screening methods(57) for oral cancer detection and for evaluation of the prevalence of OPMLs in users. This will be performed by trained dentist/dental hygienist to advise SLT and BQ users to quit all hazardous products so as to live a healthy life. Also, it will be performed in the non-users so as to encourage them to remain SLT and BQ free(33). Individuals detected with OPMLs and oral cancers will be referred for further diagnosis and treatment.

4.11 Follow up after two weeks

After 2 weeks, BCI related visual presentation will be shown to the students of intervention schools.

4.12 Follow up after twelve weeks

After 12 weeks, a follow up visit will ensue to:

1. Re-assess their perceptions regarding SLT and BQ use in users and non-users of both intervention and control clusters.
2. Re-assess their dependency on SLT and BQ in users of both intervention and control clusters.
3. Re-evaluate their perceptions (of users and non-users of intervention cluster) regarding warning labels on SLT and BQ product packet
4. Collect their quit calendars to assess quit from users of both intervention and control clusters, and
5. Collect perceived efficacy of each component of BCI that helped them quit, attempt to quit or change perceptions from all users and non-users of intervention cluster to assess efficacy of each component of BCI.

Any differences in outcome based on BCI will be assessed and documented between two arms of cluster-randomized trial.

The change in perceptions if at all, after 12 weeks, regarding imprinting of pictorial warning labels on SLT and BQ packaging will help us in recommending its implementation at National level to curb SLT & BQ use.

4.13 Plan of Analysis

4.13.1 Participants' baseline characteristics (both intervention and control arms)

Descriptive analysis will be performed for baseline characteristics regarding demographic information (e.g. age, gender, grade in which they are studying), past and present SLT and BQ use history, past smoking history in any form, and family history.

4.13.2 SLT/or BQ consumption(46) – users of both intervention and control arms

SLT/or BQ consumption will be assessed using three items:

i. Per day number of chews,
ii. Number of years since a chewer,
iii. Type of chew (areca not alone, betel quid with or without tobacco)

4.13.3 Reasons for Betel quid chewing scale(46) – users of both intervention and control arms

This validated scale is to measure the reasons for betel quid chewing. It comprises of 10-items that will be assessed on five-point scale (0=not important to 4=extremely important).

4.13.4 Betel Quid Dependence Scale (BQDS)(48) – users of both intervention and control arms

The validated BQDS is a 16-item scale which is intended to measure users' dependency on BQ. Each item on the scale marks a binary outcome (No=0 and Yes =1).

4.13.5 Fagerstörm Tobacco and Nicotine Dependency Scale (FTND-ST)(47) – users of both intervention and control arms

This modified and validated scale is a 06-item scale. The total 10 points can be scored on this scale to assess dependency on nicotine in SLT.

4.13.6 Perceptions regarding ill effects of SLT/or BQ use among adolescents (intervention arm)

There are five items that are related to behaviors amongst adolescents regarding harmful effects of SLT and BQ use in the oral cavity. All the harmful consequences are scored (No = 0 and Yes = 1) depending on participants' awareness about them. One item is based on their perception regarding warning label on SLT and BQ product pack (No = 0 and Yes = 1). Pre and post intervention differences in individual scores will be noted to assess efficacy of the BCI.

4.13.7 Quit measures

The participant's attempt in trying to quit, if it is important for them to quit, if they are confident regarding quit and if they are ready to quit, are the 4-items that will be scored (No = 0 and Yes = 1). Pre and post intervention differences in individual scores will be noted to assess efficacy of the quit component of the BCI. Along with this the quit will be assessed subjectively by quit calendars that will be submitted at the 12 weeks follow-up.

4.13.8 Oral examinations

Prevalence of OPMLs and oral cancer will be evaluated in users of SLT and BQ by conventional method of screening and test positives will be referred for further diagnosis and treatment.

The data will be a nested data thus multilevel modelling will be done to have accuracy in the analysis of the hierarchical data. Analysis of covariates will be performed to control for the potential confounders including age.

4.14 Ethical considerations

- Formal consents will be sought from schools and parents for the study.
- All relevant details will be provided to the all concerned.
- Interim analysis(58) will be performed to cater for significant differences between the two arms of the study.
- All intervention material will be provided to the control arm at the end of the study.

5 Study Time line & Schedule

S.NO	WORKING STEPS	TIME (MONTHS)																
		1	2	3	4	5	6	7	8	9	10	11	12	13	14	15	16	17
1.	Synopsis writing & approval	■	■	■	■													
2.	Grant approval	■	■	■	■													
3.	Pilot study and necessary prep.					■												
4.	Data collection						■	■	■									
5.	Data processing									■	■	■						
6.	Thesis writing/presentation												■	■	■	■	■	■

6 Appendices

Appendix 1 – Questionnaire adapted from GYTS

Appendix 2 – Questionnaire for SLT and BQ users

Appendix 3 - Fidelity Index

Appendix 4 - Behavior Changing Intervention

Appendix 5 – Informed Consent

Appendix 6 – All School Lists

7 References

1. Siddiqi K, Gupta PC, Prasad VM, Croucher R, Sheikh A. Smokeless tobacco use by south Asians. Lancet Glob Health. 2013;1(2):e71.
2. LYON F. IARC monographs on the evaluation of carcinogenic risks to humans. 2014.
3. Gupta PC, Ray CS. Smokeless tobacco and health in India and South Asia. Respirology. 2003;8(4):419-31.
4. Siddiqi K, Shah S, Abbas SM, Vidyasagaran A, Jawad M, Dogar O, et al. Global burden of disease due to smokeless tobacco consumption in adults: analysis of data from 113 countries. BMC Med. 2015;13:194.
5. Siddiqi K, Scammell K, Huque R, Khan A, Baral S, Ali S, et al. Smokeless Tobacco Supply Chain in South Asia: A Comparative Analysis Using the WHO Framework Convention on Tobacco Control. Nicotine Tob Res. 2015.
6. Sreeramareddy CT, Pradhan PM, Mir IA, Sin S. Smoking and smokeless tobacco use in nine South and Southeast Asian countries: prevalence estimates and social determinants from Demographic and Health Surveys. Popul Health Metr. 2014;12:22.
7. Mukherjee A, Sinha A, Taraphdar P, Basu G, Chakrabarty D. Tobacco abuse among school going adolescents in a rural area of West Bengal, India. Indian journal of public health. 2012;56(4):286.
8. Shrivastava N, Verma N, Bhawnani D, Soni GP. Prevalence of smokeless tobacco use among school going adolescent students of Raipur city Chhattisgarh state, India. Int J Res Med Sci. 2015;3(4):921-4.
9. Danaher BG, Severson HH, Zhu S-H, Andrews JA, Cummins SE, Lichtenstein E, et al. Randomized controlled trial of the combined effects of web and quitline interventions for smokeless tobacco cessation. Internet interventions. 2015;2(2):143-51.
10. Anwar S, Williams SA, Scott-Smith J, Sage H, Baweja S, Singal M, et al. A comparison of attitudes and practices of gutka users and non-users in Chitrakoot, India. A pilot. Primary Dental Care. 2005;12(1):5-10.
11. Sorensen G, Gupta PC, Sinha DN, Shastri S, Kamat M, Pednekar MS, et al. Teacher tobacco use and tobacco use prevention in two regions in India: qualitative research findings. Preventive medicine. 2005;41(2):424-32.
12. Croucher R, Pau AK, Jerreat M, Begum S, Marcenes W. Oral Health of Bangladeshi Women Tobacco-with-paan Users and Self-reported Oral Pain Following Tobacco Cessation. Journal of public health dentistry. 2003;63(4):235-9.
13. Kakde S, Bhopal R, Jones C. A systematic review on the social context of smokeless tobacco use in the South Asian population: implications for public health. Public health. 2012;126(8):635-45.
14. Hossain MS, Kypri K, Rahman B, Milton AH. Smokeless tobacco consumption in the South Asian population of Sydney, Australia: prevalence, correlates and availability. Drug and alcohol review. 2014;33(1):86-92.
15. Mishra S, Mishra M. Tobacco: Its historical, cultural, oral, and periodontal health association. Journal of International Society of Preventive & Community Dentistry. 2013;3(1):12.
16. Williams S, Malik A, Chowdhury S, Chauhan S. Sociocultural aspects of areca nut use. Addiction biology. 2002;7(1):147-54.
17. Ali NS, Khuwaja AK, Ali T, Hameed R. Smokeless tobacco use among adult patients who visited family practice clinics in Karachi, Pakistan. Journal of Oral Pathology & Medicine. 2009;38(5):416-21.
18. Khan A, Huque R, Shah SK, Kaur J, Baral S, Gupta PC, et al. Smokeless tobacco control policies in South Asia: a gap analysis and recommendations. Nicotine & Tobacco Research. 2014:ntu020.
19. Winstock A. Areca nut-abuse liability, dependence and public health. Addiction biology. 2002;7(1):133-8.
20. Boucher BJ, Mannan N. Metabolic effects of the consumption of Areca catechu. Addiction biology. 2002;7(1):103-10.
21. Warnakulasuriya S, Peters T. Introduction: Biology, medical and socio-economic aspects of areca nut use. Addiction biology. 2002;7(1):75-6.
22. Lin C-F, Wang J-D, Chen P-H, Chang S-J, Yang Y-H, Ko Y-C. Predictors of betel quid chewing behavior and cessation patterns in Taiwan aborigines. BMC Public Health. 2006;6(1):1.
23. Gupta P, Warnakulasuriya S. Global epidemiology of areca nut usage. Addiction biology. 2002;7(1):77-83.
24. Abbas SM, Alam AY, Usman M, Siddiqi K. Smokeless tobacco consumption in a multi-ethnic community in Pakistan: a cross-sectional study. East Mediterr Health J. 2014;20(6):385-90.
25. Bile K, Shaikh J, Afridi H, Khan Y. Smokeless tobacco use in Pakistan and its association with oropharyngeal cancer. 2010.
26. Guha N, Boffetta P, Wünsch Filho V, Neto JE, Shangina O, Zaridze D, et al. Oral health and risk of squamous cell carcinoma of the head and neck and esophagus: results of two multicentric case-control studies. American journal of epidemiology. 2007;166(10):1159-73.
27. Khan Z, Tönnies J, Müller S. Smokeless tobacco and oral cancer in South Asia: a systematic review with meta-analysis. Journal of cancer epidemiology. 2014;2014.
28. Hospital SKMC. Shaukat Khanum Memorial Cancer Hospital Registry 2014 [Cited on February 13, 2016].
29. Nelson DE, Mowery P, Tomar S, Marcus S, Giovino G, Zhao L. Trends in smokeless tobacco use among adults and adolescents in the United States. American Journal of Public Health. 2006;96(5):897-905.
30. Kakde S, Bhopal RS, Jones CM. A systematic review on the social context of smokeless tobacco use in the South Asian population: Implications for public health. Public Health. 2012;126(8):635-45.
31. Ebbert J, Montori VM, Erwin PJ, Stead LF. Interventions for smokeless tobacco use cessation. Cochrane Database Syst Rev. 2011;2.
32. Stevens VJ, Severson H, Lichtenstein E, Little SJ, Leben J. Making the most of a teachable moment: a smokeless-tobacco cessation intervention in the dental office. American Journal of Public Health. 1995;85(2):231-5.
33. Gansky SA, Ellison JA, Rudy D, Bergert N. Cluster-randomized controlled trial of an athletic trainer-directed spit (smokeless) tobacco intervention for collegiate baseball athletes: results after 1 year. Journal of athletic training. 2005;40(2):76.

34. Walsh MM, Hilton JF, Ellison JA, Gee L, Chesney MA, Tomar SL, et al. Spit (smokeless) tobacco intervention for high school athletes: results after 1 year. Addictive behaviors. 2003;28(6):1095-113.
35. Ebbert JO, Elrashidi MY, Stead LF. Interventions for smokeless tobacco use cessation. status and date: New search for studies and content updated (conclusions changed), published in. 2015(10).
36. NICE. Smokless Tobacco Cessation - South Asian Communities (PH39). Available from: http://guidance.nice.org.uk/PH39.
37. Croucher R, Shanbhag S, Dahiya M, Kassim S, Csikar J, Ross L. Smokeless tobacco cessation in South Asian communities: a multi-centre prospective cohort study. Addiction. 2012;107 Suppl 2:45-52.
38. Sinha DN, Abdulkader RS, Gupta PC. Smokeless tobacco-associated cancers: A systematic review and meta-analysis of Indian studies. Int J Cancer. 2016;138(6):1368-79.
39. Michie S, Hyder N, Walia A, West R. Development of a taxonomy of behaviour change techniques used in individual behavioural support for smoking cessation. Addictive behaviors. 2011;36(4):315-9.
40. Michie S, Richardson M, Johnston M, Abraham C, Francis J, Hardeman W, et al. The behavior change technique taxonomy (v1) of 93 hierarchically clustered techniques: building an international consensus for the reporting of behavior change interventions. Annals of behavioral medicine. 2013;46(1):81-95.
41. Kriemler S, Meyer U, Martin E, Van Sluijs E, Andersen L, Martin B. Effect of school-based interventions on physical activity and fitness in children and adolescents: a review of reviews and systematic update. British journal of sports medicine. 2011;45(11):923-30.
42. Pakistan Buraeu of Statistics GoP. Distribution of districts. http://www.pbscensus.gov.pk.
43. Ali TS, Mogren I, Krantz G. Intimate partner violence and mental health effects: A population-based study among married women in Karachi, Pakistan. International journal of behavioral medicine. 2013;20(1):131-9.
44. fact sheets Pakistan—Karachi C. Global Youth Tobacco Survey (GYTS)[Internet]. Geneva: World Health Organization. 2010.
45. Bennett S, Woods T, Liyanage WM, Smith DL. A simplified general method for cluster-sample surveys of health in developing countries. World Health Stat Q. 1991;44(3):98-106.
46. Little MA, Pokhrel P, Murphy KL, Kawamoto CT, Suguitan GS, Herzog TA. The reasons for betel-quid chewing scale: assessment of factor structure, reliability, and validity. BMC oral health. 2014;14(1):62.
47. Ebbert JO, Patten CA, Schroeder DR. The Fagerström test for nicotine dependence-smokeless tobacco (FTND-ST). Addictive behaviors. 2006;31(9):1716-21.
48. Herzog TA, Murphy KL, Little MA, Suguitan GS, Pokhrel P, Kawamoto CT. The Betel Quid Dependence Scale: replication and extension in a Guamanian sample. Drug and alcohol dependence. 2014;138:154-60.
49. Craig P, Dieppe P, Macintyre S, Michie S, Nazareth I, Petticrew M. Developing and evaluating complex interventions: the new Medical Research Council guidance. Bmj. 2008;337:a1655.
50. Trakroo A, Sunil M, Trivedi A, Garg R, Kulkarni A, Arora S. Efficacy of Oral Brush Biopsy without Computer-Assisted Analysis in Oral Premalignant and Malignant Lesions: A Study. Journal of international oral health: JIOH. 2015;7(3):33.
51. McEwen A. Standard Treatment Programme: A Guide to Behavioural Support for Smoking Cessation. UK: Department of Health, National Centre for Smoking Cessation and Training; 2014.
52. Malik MA, Hassan R. An Analysis of Parallel Education Systems in Pakistan, and the Challenges They Pose in Education Research. Advances in Social Sciences Research Journal. 2015;2(10).
53. Kostova D, Dave D. Smokeless tobacco use in India: Role of prices and advertising. Social Science & Medicine. 2015;138:82-90.
54. Lane KL, Royer DJ, Messenger ML, Common EA, Ennis RP, Swogger ED. Empowering teachers with low-intensity strategies to support academic engagement: implementation and effects of instructional choice for elementary students in inclusive settings. Education and Treatment of Children. 2015;38(4):473-504.
55. Abry T, Hulleman CS, Rimm-Kaufman SE. Using indices of fidelity to intervention core components to identify program active ingredients. American Journal of Evaluation. 2015;36(3):320-38.
56. Bond G, Williams J, Evans L, Salyers M, Kim H, Sharpe H, et al. Psychiatric rehabilitation fidelity toolkit. Cambridge, MA: Human Services Research Institute. 2000.
57. Rashid A, Warnakulasuriya S. The use of light-based (optical) detection systems as adjuncts in the detection of oral cancer and oral potentially malignant disorders: a systematic review. Journal of Oral Pathology & Medicine. 2015;44(5):307-28.
58. Pocock SJ. Interim analyses for randomized clinical trials: the group sequential approach. Biometrics. 1982:153-62.

Appendix 1

Behavior Changing Intervention for smokeless tobacco and betel quid use in adolescents: A cluster-randomized trial

Questionnaire 1 – Demographic data and Habit related Questions (adapted from Global Youth Tobacco Survey) – to be filled by all clusters (control and intervention inclusive of users and non-users)

Code: _______________
Enrolment #: _________
Date: _______________

1. **How old are you?**
   a. 11-year-old or younger
   b. 12-year-old
   c. 13-year-old
   d. 14-year-old
   e. 15-year-old
   f. 16-year-old
   g. 17-year-old or older

2. **What is your gender?**
   a. Male
   b. Female

3. **In what grade/class are you?**
   a. VI
   b. VII
   c. VIII
   d. IX
   e. X

4. **During an average week, how much money (PKR) do you have that you can spend on yourself, however you want?**
   a. 100- 200
   b. 201- 300
   c. 301- 400
   d. 401- 500
   e. More than 500, specify: _________________

5. **Do your parents work?**
   a. Father only
   b. Mother only
   c. Both
   d. Neither
   e. Don't know

6. **What level of education did your father complete?**
   a. Primary
   b. Middle
   c. Matric
   d. Inter
   e. Graduation
   f. Masters
   g. Don't know

7. **What level of education did your mother complete?**
   a. Primary
   b. Middle
   c. Matric
   d. Inter
   e. Graduation
   f. Masters
   g. Don't know

8. **What do you think you will be doing when you finish high school?**
   a. Doctor
   b. Engineer
   c. Pilot
   d. Teacher
   e. Nurse
   f. Solider
   g. Don't know

Smokeless tobacco, betel quid with or without tobacco

The next questions ask about smokeless tobacco. This includes chewing tobacco such as: tobacco leaf, tobacco leaf and lime-khaini/ sada/ surti, gutka, panmasala with zarda or pan; betel quid, chalia, applying tobacco such

as gul, gudaku, mishri/20asher/ tapkir, tuibur, tobacco tooth paste-dentobac etc. tobacco tooth powder-lal dantmanjan, etc.; snuff such as nas and naswar.

9. Do you use any of the following products?

a. Paan (betel quid)
b. Paan masala (chalia.betel nut)
c. Zarda
d. Ghutka
e. Naswar
f. Others ___________________, please specify

10. Have you used any chewing tobacco, pan, chalia, naswar and gutka (or any form of SLT) at least once in past 30 days?

a. Yes
b. No
c. I don't know

11. Does any of your closest friend chew tobacco, eat pan, chalia, naswar, gutka or any other similar product?

a. None of them
b. Some of them
c. Most of them
d. All of them

12. Does any of your teacher chew tobacco, eat pan, chalia, naswar, gutka or any other similar product?

a. None of them
b. Some of them
c. Most of them
d. All of them

13. Do your parents chew tobacco, eat pan, chalia, naswar, gutka or any other similar product?

a. None of them
b. Some of them
c. Most of them
d. All of them

14. During the past 12 months, did you discuss in any of your classes the reasons why people your age use chewing tobacco, pan, chalia, naswar and gutka (or any form of SLT)?

a. Yes
b. No
c. Not sure

15. Do you think chewing tobacco, pan, chalia, naswar and gutka (or any form of SLT) are harmful to your health?

a. Definitely not
b. Probably not
c. Probably yes
d. Definitely yes

16. During the past 12 months, were you taught in any of your classes about the effects of chewing tobacco, pan, chalia, naswar and gutka (or any form of SLT) like they make your teeth yellow, cause tooth loss, cause white or red patch in your mouth, cause burning in mouth, cause ulcers in mouth, cause mouth cancer, or make you smell bad?

a. Yes
b. No
c. Not sure

17. Do you know chewing tobacco, pan, chalia, naswar and gutka (or any form of SLT) can cause stained teeth?

a. Yes
b. No

18. Do you know chewing tobacco, pan, chalia, naswar and gutka (or any form of SLT) can cause oral ulcers?

a. Yes
b. No

19. Do you know chewing tobacco, pan, chalia, naswar and gutka (or any form of SLT) can cause white patch that can lead to cancer?

a. Yes
b. No

20. Do you know chewing tobacco, pan, chalia, naswar and gutka (or any form of SLT) can cause limited mouth opening which in turn may cause cancer?

a. Yes
b. No

21. Do you know chewing tobacco, pan, chalia, naswar and gutka (or any form of SLT) can cause cancer?

a. Yes
b. No

22. During the past 12 months, did you ever try to stop chewing tobacco, pan, chalia, naswar and gutka (or any form of SLT)?

a. I have never used smokeless tobacco
b. I did not use smokeless tobacco during the past 12 months
c. Yes
d. No

23. Do you think it is important for you to stop chewing tobacco, pan, chalia, naswar and gutka (or any form of SLT)?

a. I have never used smokeless tobacco
b. I don't use smokeless tobacco now
c. Yes
d. No

24. If one of your best friends offered you chewing tobacco, pan, chalia, naswar and gutka (or any form of SLT), would you use it?

a. Definitely not
b. Probably not
c. Probably yes
d. Definitely yes

25. **Do you get to buy chewing tobacco, pan, chalia, naswar and gutka (or any form of SLT) from school canteen?**
   a. Yes
   b. No

26. Do you get to buy chewing tobacco, pan, chalia, naswar and gutka (or any form of SLT) from outside school hawkers?
   a. Yes
   b. No

27. Do you know you can save money by quitting chewing tobacco, pan, chalia, naswar and gutka (or any form of SLT)?
   a. Yes
   b. No

28. Do you know you can live longer if you quit chewing tobacco, pan, chalia, naswar and gutka (or any form of SLT)?
   a. Yes
   b. No

29. Do you know you can have a better quality of life if you quit chewing tobacco, pan, chalia, naswar and gutka (or any form of SLT)?
   a. Yes
   b. No

30.Do you know you can stay with your loved ones for long if you quit chewing tobacco, pan, chalia, naswar and gutka (or any form of SLT)?
   a. Yes
   b. No

31.Are you confident about quitting chewing tobacco, pan, chalia, naswar and gutka (or any form of SLT)?
   a. Yes
   b. No
   c. I don’t know

32.Are you ready to quit?
   a. Yes
   b. No
   c. I don’ know

33.If you see cancer picture on your product packet, will you still use it??
   a. Yes
   b. No
   c. I don’ know

34.The next questions ask about your use of tobacco. Have you ever tried or experimented with cigarette smoking, even one or two puffs?
   a. Yes
   b. No

35.Have you ever tried or experimented with shisha smoking, even one or two puffs?
   a. Yes
   b. No

Appendix 2

Behavior Changing Intervention for smokeless tobacco and betel quid use in adolescents: A cluster-randomized trial

Questionnaire 2 – Dependency and RBCS – to be filled by all clusters (control and intervention inclusive of users only)

Betel Quid Dependence Scale

Factor 1: Physical and psychological urgent need

1 Can't go on without betel nut/quid

a. Yes
b. No

2 Difficulty concentrating after reducing use

a. Yes
b. No

3 Experienced depression or drowsiness

a. Yes
b. No

4 Strong craving after reducing/stopping to chew

a. Yes
b. No

5 Spend time to find when not available

a. Yes
b. No

6 Travel great distance to find when not available

a. Yes
b. No

i. Felt agitated, irritated, or anxious after reducing

a. Yes
b. No

Factor 2: Increasing dose

ii. Trouble stopping once started chewing

a. Yes
b. No

iii. Ever chewed non-stop

a. Yes
b. No

10 Increased the amount of use after first use

a. Yes
b. No

11 Felt the need to increase amount of use periodically

a. Yes
b. No

12 Often chewed betel nut/quid more than expected

a. Yes
b. No

Factor 3: Maladaptive use

13 Continue chewing after teeth loosen or wiggle

a. Yes
b. No

14 Continue chewing if you had sensitive teeth

a. Yes
b. No

15 Continue chewing if experienced mouth ulcers

a. Yes
b. No

16 Reduced or given up activities because of chewing

a. Yes
b. No

The Fagerström Test for Nicotine Dependence-Smokeless Tobacco (FTND-ST)

Item	Answers	Points
1. How soon after you wake up to do you place your first dip?	Within 5 min	3
	6–30 min	2
	31–60 min	1
	After 60 min	0
2. How often do you intentionally swallow tobacco juice?	Always	2
	Sometimes	1
	Never	0
3. Which chew would you hate to give up most?	The first one in the morning	1
	Any other	0
4. How many cans/pouches per week do you use?	More than 3	2
	2–3	1
	1	0
5. Do you chew more frequently during the first hours after awakening than during the rest of the day?	Yes	1
	No	0

Betel quid consumption

Betel quid consumption was assessed through three items: number of chews per day, number of years as a chewer, and the type of betel quid (i.e., areca nut alone, betel quid without tobacco, or betel quid with tobacco)

1. How many numbers of chews per day you do?
2. Since how many years you have been chewing?
3. What type of SLT/or BQ you chew?
    a. Areca nut alone
    b. Betel quid without tobacco
    c. Betel quid with tobacco
    d. Any other

Reasons for betel-quid chewing scale (RBCS)

Reinforcement construct

1. I like the taste
    a. 0 = NOT IMPORTANT
    b. 1= SOME WHAT IMPORTANT
    c. 2= NEUTRAL
    d. 3= IMPORTANT
    e. 4=EXTRMELY IMPORTANT
2. I like to have something in my mouth at all times
    a. 0 = NOT IMPORTANT
    b. 1= SOME WHAT IMPORTANT
    c. 2= NEUTRAL
    d. 3= IMPORTANT
    e. 4=EXTRMELY IMPORTANT

Social/cultural construct

3. All of my friends chew
    a. 0 = NOT IMPORTANT
    b. 1= SOME WHAT IMPORTANT
    c. 2= NEUTRAL
    d. 3= IMPORTANT
    e. 4=EXTRMELY IMPORTANT

4. My family members chew
    a. 0 = NOT IMPORTANT
    b. 1= SOME WHAT IMPORTANT
    c. 2= NEUTRAL
    d. 3= IMPORTANT
    e. 4=EXTRMELY IMPORTANT

5. It’s rude not to chew
    a. 0 = NOT IMPORTANT
    b. 1= SOME WHAT IMPORTANT
    c. 2= NEUTRAL
    d. 3= IMPORTANT
    e. 4=EXTRMELY IMPORTANT

6. People will not respect me if I don’t chew
    a. 0 = NOT IMPORTANT
    b. 1= SOME WHAT IMPORTANT
    c. 2= NEUTRAL
    d. 3= IMPORTANT
    e. 4=EXTRMELY IMPORTANT

Stimulation construct

7. It relaxes me
    a. 0 = NOT IMPORTANT
    b. 1= SOME WHAT IMPORTANT
    c. 2= NEUTRAL
    d. 3= IMPORTANT
    e. 4=EXTRMELY IMPORTANT

8. It gives me energy
    a. 0 = NOT IMPORTANT
    b. 1= SOME WHAT IMPORTANT
    c. 2= NEUTRAL
    d. 3= IMPORTANT
    e. 4=EXTRMELY IMPORTANT

9. It helps me make decisions
    a. 0 = NOT IMPORTANT
    b. 1= SOME WHAT IMPORTANT
    c. 2= NEUTRAL
    d. 3= IMPORTANT
    e. 4=EXTRMELY IMPORTANT

10. I like the way it makes me feel
    a. 0 = NOT IMPORTANT
    b. 1= SOME WHAT IMPORTANT
    c. 2= NEUTRAL
    d. 3= IMPORTANT
    e. 4=EXTRMELY IMPORTANT

Appendix 3

Quit Calendar

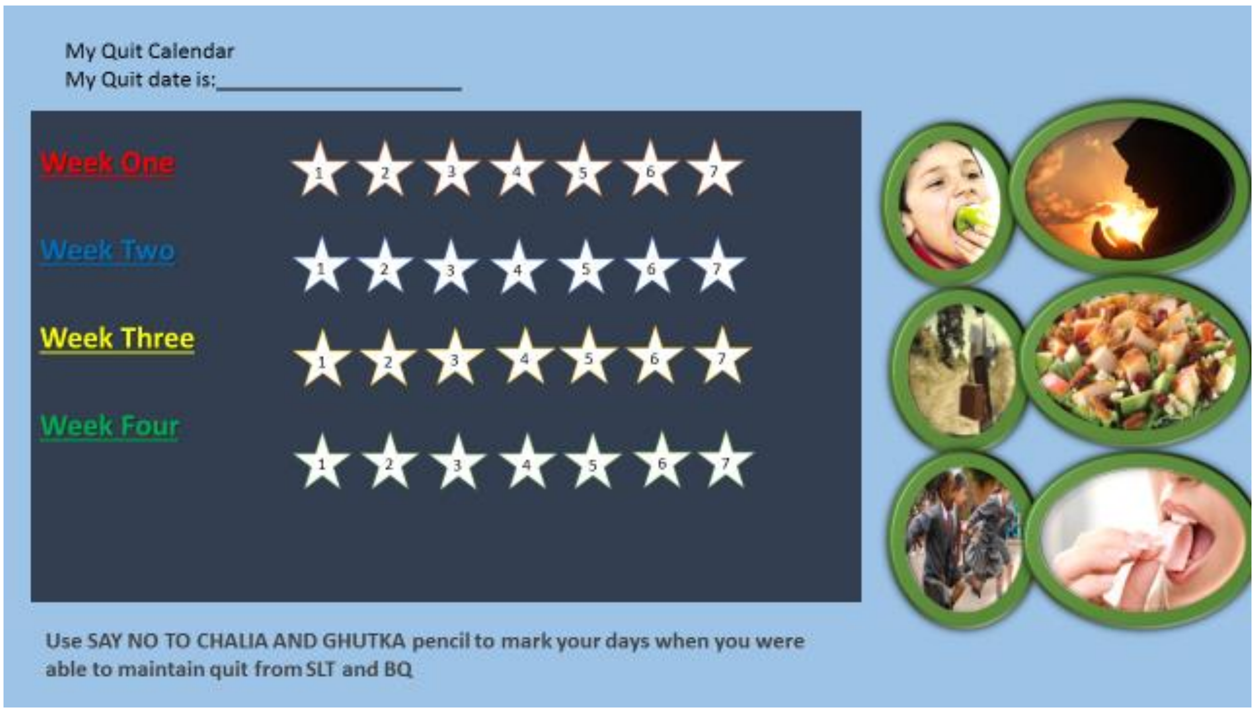

Appendix 4

Consent Form

To

The Parent Name of the school Karachi

Subject: Permission for a Behavioral Intervention in your school regarding Smokeless Tobacco and Betel Quid chew

Respected Sir/Madam

It is my privilege to inform you that we are conducting a very large and comprehensive Behavioral intervention for adolescents to curb their habit of Smokeless Tobacco (SLT) and Betel Quid (BQ) chew and reduce resultant Oral Cancer incidence in our population.

For the same, 26 schools will be selected from throughout Karachi and your child has been selected from his/her school to participate in this study. I wish to request you to grant permission so we may intervene to change perceptions your child regarding SLT/or BQ chew regardless of the fact whether s/he is a user or non-user.

All the relevant details are attached with this invitation letter. There are no harmful effects of the study. We will also do free full mouth check-ups of your child and provide you with the reports.

Parents are encouraged to me by leaving at text on given contact number and you will be contacted by the me within 24 hours to answer all your queries regarding this study.

Kindly sign this and return it to school administration within (07) days,

I hereby give consent that my child may participate in this healthy activity.

Parent/guardian's Signature: ________________________

If due to any reason you do not wish to join hands in this endeavor, kindly inform us at the given contact number or email ID within 03 days of receipt of this invitation.

We look forward to your positive response in this regard. Let's join hands in efforts to make our region SMOKELESS TOBACCO FREE.

Best Regards

____

Prof. Dr. Azmina Hussain (PI) BDS,
MPhil (Microbiology), PhD Scholar (Public Health)
Professor & Head-Oral Pathology
DOW UNIVERSITY OF HEALTH SCIENCES
Email: azmina.hussain@duhs.edu.pk
Contact: +92-336-2332763

To
The Principal/Head of the school
Name of the school
Karachi

Subject: Permission for a Behavioral Intervention in your school regarding Smokeless Tobacco and Betel Quid chew

Respected Sir/Madam

It is my privilege to inform you that we are conducting a very large and comprehensive Behavioral intervention for adolescents to curb their habit of Smokeless Tobacco (SLT) and Betel Quid (BQ) chew and reduce resultant Oral Cancer incidence in our population. For the same, 26 schools will be selected from throughout Karachi and your school has been shortlisted. I wish to request you to grant permission so we may intervene to change perceptions of grade VI-X adolescents regarding SLT/or BQ chew. All the relevant details are attached with this invitation letter. It is requested from school's administration to kindly seek consent from parents of students studying in grade VI-X by sending them consent forms(attached) and return within (07) working days. Parents are encouraged to contact PI by leaving at text on given contact number and they will be contacted by the same within 24 hours to answer their queries regarding this study. If due to any reason you do not wish to join hands in this endeavor, kindly inform us at the given contact number or email ID within 03 days of receipt of this invitation. The confidentiality of the information provided will be maintained. We look forward to your positive response in this regard. Let's join hands in efforts to make our region SMOKELESS TOBACCO FREE.

Best Regards

____

Prof. Dr. Azmina Hussain (PI)
BDS, MPhil (Microbiology), PhD Scholar (Public Health)
Professor & Head-Oral Pathology
In charge- School of Dental Care Professionals
DOW UNIVERSITY OF HEALTH SCIENCES
Email: azmina.hussain@duhs.edu.pk
Contact: +92-336-2332763
